# Caesarean section and risk of infection in offspring: systematic review and meta-analysis of observational studies

**DOI:** 10.1136/bmjmed-2024-000995

**Published:** 2024-11-27

**Authors:** Isobel Masson Francis Todd, Maria Christine Magnus, Lars Henning Pedersen, David Burgner, Jessica Eden Miller

**Affiliations:** 1 Infection and Immunity, Murdoch Children's Research Institute, Parkville, VIC, Australia; 2 Department of Paediatrics, The University of Melbourne, Melbourne, VIC, Australia; 3 Centre for Fertility and Health, Norwegian Institute of Public Health, Oslo, Norway; 4 Clinical Medicine, Aarhus University, Aarhus, Denmark; 5 Obstetrics and Gynecology, Aarhus University Hospital, Aarhus, Denmark; 6 Infectious Diseases, Royal Children's Hospital Melbourne Department of General Medicine, Parkville, Victoria, Australia

**Keywords:** Respiratory tract infections, Obstetrics

## Abstract

**Objective:**

To compare the risk of hospital admissions with infections and infections not in hospital in children born by caesarean section with children born by vaginal birth.

**Data sources:**

Medline, Embase, and PubMed were searched with no restriction on start date up to 12 February 2024.

**Study selection:**

Observational studies were included that reported the association between caesarean section and vaginal birth in relation to the risk of infections (both those that lead to hospital admission and those that do not) up to 18 years of age. Studies were excluded if they were not representative of a general population or if they focused on congenital, neonatal, or vertically acquired infections. No restrictions were made for language, publication date, or setting.

**Review methods:**

Findings for hospital admissions with infection were synthesised by meta-analyses of specific infection outcomes and type of caesarean birth (emergency *v* elective) and findings for other infections (ie, infection episodes reported by parents and primary care visits) by direction of effect. Risk of bias was assessed using the ROBINS-E tool and the overall certainty of evidence through the GRADE framework.

**Results:**

31 eligible studies of over 10 million children were included. Findings were from population-based birth cohorts and registry data linkage studies in high income countries. Cohort sizes ranged from 288 to 7.2 million and follow up age was from one to 18 years. Outcomes included overall and specific clinical categories of infection. From studies of overall admission to hospital with infection, the proportion of children admitted ranged between 9-29% across exposure groups. In random-effects meta-analyses combining hazard ratios, children delivered by caesarean section had an increased rate of hospital admission with infections overall and in three common clinical infection categories: (1) overall admissions to hospital with infection (emergency caesarean section: n=6 study populations, hazard ratio 1.10 (95% confidence interval 1.06 to 1.14), τ^2^=0.0009, I^2^=96%; elective caesarean section: n=7, 1.12 (1.09 to 1.15), τ^2^=0.0006, I^2^=88%); (2) admission to hospital for upper respiratory infections (emergency caesarean section: n=7, 1.11 (1.09 to 1.13), τ^2^=0.0003, I^2^=73%; elective caesarean section: n=7, 1.16 (1.12 to 1.20), τ^2^=0.0012, I^2^=89%); (3) admission to hospital for lower respiratory infections (emergency caesarean section: n=8, 1.09 (1.06 to 1.12), τ^2^=0.0010, I^2^=88%; elective caesarean section: n=8, 1.13 (1.10 to 1.16), τ^2^=0.0009, I^2^=84%); (4) admission to hospital for gastrointestinal infections (emergency caesarean section: n=7, 1.19 (1.13 to 1.26), τ^2^=0.0025, I^2^=86%; elective caesarean section: n=7, 1.20 (1.15 to 1.25), τ^2^=0.0009, I^2^=67%). Eight of 11 studies of other infections suggested an increased risk of their primary infection outcome in those born by caesarean section. Risk of bias concerns primarily related to confounding.

**Conclusions:**

Findings from high income countries showed a consistent association between caesarean section birth and greater risk of infections in children across various settings. Limitations of existing studies include the potential for unmeasured confounding, specifically confounding by indication, and a scarcity of studies from low and middle income countries.

**Review registration:**

PROSPERO (CRD42022369252).

**WHAT IS ALREADY KNOWN ON THIS TOPIC**

- Many studies have reported associations between caesarean section birth and various infection outcomes across childhood but to date, this research has not been systematically combined

**WHAT THIS STUDY ADDS**

- This systematic review and meta-analysis investigated 31 observational studies of over 10 million children
- Caesarean section birth was associated with increased risks of overall and specific types of infection in hospital and outside of hospital throughout childhood

**HOW THIS STUDY MIGHT AFFECT RESEARCH, PRACTICE, OR POLICY**

- While caesarean section is often medically indicated, potential increased risk of childhood infection should be considered alongside other maternal and offspring considerations

## Introduction

 The proportion of births by caesarean section worldwide has increased from around 12% to over 20% in the past 20 years.^1^ This proportion varies across regions of the world with a range of 5–43%.^2^

Numerous observational studies have investigated associations between caesarean section and short and long term outcomes in offspring, including neonatal respiratory disorders,^3^ neurodevelopmental outcomes,^4 5^ overweight, and obesity.^6^,^9^ The postnatal microbiome is markedly different following caesarean section with persistent effects on immune responses^10 11^; the associations between mode of birth and outcomes related to the immune system—allergy, autoimmune diseases, and infection—are therefore of particular interest. Studies of the risk of allergic and autoimmune diseases in offspring according to mode of birth have been summarised in previous systematic reviews.^12^,^17^

Mode of birth and infection has also been examined in previous systematic reviews in relation to specific types of infection (eg, vertically acquired infections and respiratory infections) often in the context of several other childhood outcomes.^14 18 19^ Infection is a leading cause of childhood morbidity and a major cause of mortality in low and middle income countries,^20^ but no comprehensive synthesis has been done of the data regarding mode of birth and childhood infection that examines various levels of infection severity and comparing clinical infection categories. Synthesis of observational data are of particular importance in relation to mode of birth where a randomised controlled trial is unethical in most circumstances. We therefore aimed to undertake a systematic review and meta-analysis of observational studies comparing caesarean section birth to vaginal birth in relation to the risk in children of hospital admission for infection and infection without hospital admission. We considered subtypes of caesarean delivery (eg, emergency and elective) because of varying hypotheses on the biological mechanisms, particularly related to acquisition of the maternal microbiome, through which mode of birth may affect offspring health,^21 22^ and included both overall and specific clinical infection categories.

## Methods

We registered the protocol for this systematic review on the International Prospective Register of Systematic Reviews^23^ (PROSPERO) prior to title and abstract screening (PROSPERO identifier CRD42022369252) (online supplemental appendix 1 for protocol and deviations). This report follows the Preferred Reporting Items for Systematic Reviews and Meta-Analysis (PRISMA) guidelines^24^ (online supplemental appendix 2).

### Literature search and eligibility criteria

We included peer reviewed studies that compared the risk of infections in the first 18 years of life between children born by caesarean section and those born by vaginal birth. Original observational studies using a case control, cohort (follow up or registry based), or cross-sectional design were considered. We excluded studies with samples not based on the general population; where the study group was selected for a specific condition or characteristic of interest (eg, preterm birth) rather than recruitment from a general population. This design was to reduce heterogeneity where effects may differ among subgroups with specific characteristics and potential confounding by indication.

We considered any studies where caesarean birth was compared with vaginal birth. This included different categorisations of caesarean births (eg, any, emergency or acute, elective or planned, with or without labour, and on maternal request) and of vaginal births (eg, any, non-instrumental or instrumental, with or without induction).

Infection outcomes could be any level of severity: from infections in ambulatory care or outside of hospital (through self-report or doctor diagnosis) through to admission to hospital with infection. We also included infection outcomes of any level of specificity: (1) single pathogens (eg, respiratory syncytial virus and influenza), (2) specific syndromes (eg, bronchiolitis and pneumonia), (3) common clinical infection categories (eg, respiratory and gastrointestinal), and (4) overall or general infection morbidity. We excluded studies where the outcome was prevalent rather than incident cases of infections; vertically acquired infections; or neonatal infections. Vertically acquired and neonatal infections were excluded to capture longer term effects of mode of birth and to reduce confounding by indication (ie, perinatal infections impacting iatrogenic decisions regarding mode of birth).

We identified studies by searching Medline and Embase via Ovid, and PubMed, from database conception to present. This search was developed in consultation with a health librarian and used both MeSH and subject headings in addition to keywords, synonyms, and common misspellings. The search was developed in Medline and translated into Embase and PubMed with the librarian's assistance. The full search strategy is presented in online supplemental appendix 3. We tested the sensitivity of our search by ensuring that all thirteen studies that we had prior knowledge of were identified before proceeding with title and abstract screening. We did not restrict by language, publication date, or location. We conducted the initial search on the 18 October 2022, a forwards and backwards citation search on the 30 October 2023 for the studies that met our inclusion criteria from the first round of screening, and a final search on the 12 February 2024.

Search results were imported to the systematic review management software Covidence,^25^ and duplicates were removed. Two reviewers (IMFT and JEM) independently screened the title and abstracts of the search results for eligibility. Full texts were retrieved and considered against the inclusion criteria independently by the same reviewers. At each stage, discrepancies were resolved by consensus or by referring to a third author (DB).

### Data extraction and outcome assessments

Data from each study were extracted by one reviewer (IMFT) and verified by a second reviewer (JEM) using a piloted form which collected information on the study population, design, statistical analysis, and findings. Data included in the review were all available in the published papers and accompanying supplementary material except for one publication^26^ where we contacted authors to provide separate estimates for elective and emergency caesarean.

### Risk of bias and certainty of evidence assessment

We evaluated the risk of bias for each study using the risk of bias in non-randomised studies of exposure (ROBINS-E) tool (launch version released in July 2022).^27^ This tool assesses risk of bias across seven domains using a decision problem to determine the risk of bias in each domain. Based on our causal model (online supplemental figure 1), we prespecified four factors—maternal age, maternal smoking during pregnancy, socioeconomic status, and at least one maternal pregnancy or health-related risk factor for caesarean section—as important confounders requiring adjustment, in the preliminary assessment of the ROBINS-E tool. Two reviewers (IMFT, JEM) independently completed the risk of bias assessment across the seven domains and the overall risk of bias assessment for each study. Disagreements were resolved through discussion to reach consensus. Certainty of evidence was assessed using the grades of recommendation, assessment, development, and evaluation (GRADE) framework.^28^

### Data synthesis and analysis

The studies were grouped by whether the infection outcome was admission to hospital or other infections (parent reported episodes of infection, primary care diagnoses, emergency department presentations, and pathogen specific registries). For studies where the infection outcome did not align with other studies, we present the findings but did not include the result in a meta-analysis. Due to overlapping study populations across studies, we considered several study features to determine which results to statistically synthesise in the meta-analyses, according to the following hierarchy: (1) studies with the largest sample size; (2) studies with the broadest infection outcome (eg, lower respiratory infections preferred over respiratory syncytial virus); (3) similarity of the effect measure type (eg, odds, hazard, or risk ratio), because the odds ratio is not an appropriate estimate of the risk ratio for non-rare outcomes; (4) similarity by exposure grouping; and (5) if overlapping study populations were still present, studies with more similar follow-up age were grouped in the main meta-analysis and we then performed sensitivity analyses using the effect estimate from the alternate study. As a result of this hierarchy, we were able to perform meta-analyses using the reported hazard ratios for elective or planned caesarean section and emergency or acute caesarean section separately for four infection outcomes that led to admission to hospital: overall hospital admission with infection and three common clinical infection categories (upper respiratory, lower respiratory, and gastrointestinal infections).

All data synthesis used the adjusted effect estimates and confidence intervals from the primary studies. Meta-analyses used a random-effects model and the restricted maximum likelihood estimator of τ^2^ to estimate the pooled hazard ratio.^29^ Confidence intervals and prediction intervals were derived using the Hartung-Knapp method.^30 31^ A random effects model was chosen to account for variability in study designs. For each meta-analysis, we report the τ^2 ^statistic as a measure of the between-study variance and the I^2^ as the proportion of total variance attributable to between-study heterogeneity. Publication bias was not assessed in our meta-analyses because all analyses included fewer than ten studies.^32^ We used R statistical software version 4.3.2 to conduct statistical analyses and produce figures.

### Patient and public involvement

As this was a systematic review and meta-analysis of previously published research, no patients or public were involved in the design of this study.

## Results

### Study selection and characteristics

**Figure 1:**
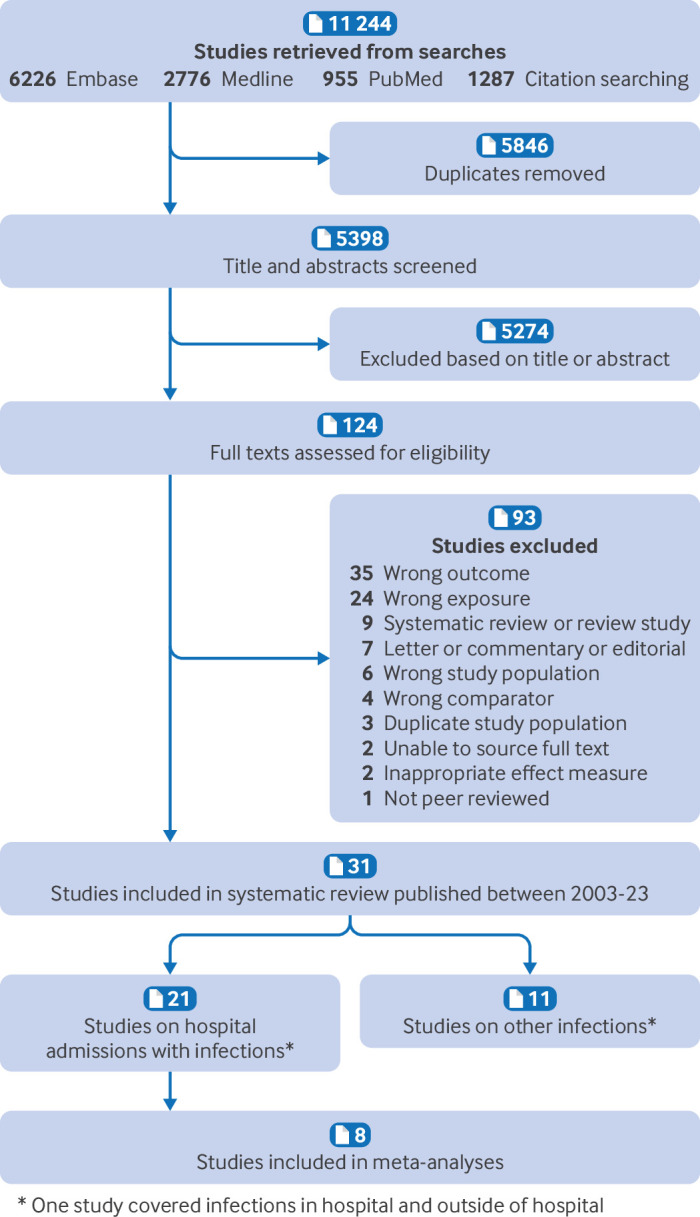
Literature flowchart

Our initial and updated search retrieved 5398 studies after excluding duplicates. Following title and abstract screening, 124 studies remained for full text screening. Of these, 32 studies met the inclusion criteria. One study^33^ was removed as the sample and outcome were identical to another study^34^; leaving 31 studies in the systematic review (figure 1) of which 8 were included in meta-analyses. The 31 studies included 22 registry based cohorts, eight cohort studies, and one study with both. The characteristics of the 31 studies, including the mode of birth categories and primary infection outcomes, are summarised in table 1. Ten of the study populations were from Scandinavia,^34^,^43^ three from North America,^26 44 45^ nine from Australia,^46^,^54^ three from the United Kingdom,^55^,^57^ two from Israel,^58 59^ one from Germany,^60^ one from China,^61^ and two multinational studies,^62 63^ one of which included data from five countries. From these 31 studies, 18 distinct study populations were remaining after accounting for overlapping samples. Sample size ranged from 288 to 7.2 million. Follow up started at birth in all but three studies^38 44 61^ and continued until between one to 18 years of age. All identified studies were published in English.

**Table 1:** Characteristics of included studies

Reference	Country	Study design	Years	Follow-up age (years)*	Cohort size	Main exposure categorisation	Primary infection outcome†
**Hospital admission with infection**							
Auger 2021^26^	Canada	Registry cohort	2006-19	13	731 803	Non-instrumental vaginal (reference), Any caesarean	Hospital admission with infection
Bentley 2018^46^	Australia	Registry cohort	2007-14	5	488 603	Vaginal spontaneous labour (reference), Caesarean pre-labour, Spontaneous labour caesarean, Labour induced caesarean	Hospital admission with infection
Essa 2020^58^	Israel	Registry sibling study	1991-2014	18	13 516	Any caesarean	Hospital admission with infection
Wainstock 2019^59^	Israel	Registry cohort	1991-2014	18	138 910	Elective caesarean	Hospital admission with infection
Miller 2020^62^	Denmark, UK, Australia	Registry cohort	1996-2015	5	7.2 million	Any caesarean‡	Hospital admission with infection
Christensen 2018^35^§	Denmark	Cohort	2010-15	5	2431	Emergency caesarean, Elective caesarean	Hospital admission with infection and infections at home
Alterman 2021^55^	UK	Cohort & registry cohort	2000-17	1	407 725	Emergency caesarean, Elective caesarean	Hospital admission with upper and lower respiratory infections
Moore 2010^47^	Australia	Registry cohort	1996-2005	2	244 563	Non-instrumental vaginal (reference), Emergency caesarean, Elective caesarean	Hospital admission with lower respiratory infections
Haataja 2020^36^	Finland	Registry cohort	1991-2009	7	948 695	Emergency caesarean, Elective caesarean	Hospital admission with pneumonia and bronchitis or bronchiolitis
Moore 2012^48^	Australia	Registry cohort	1996-2005	2	212 068	Vaginal spontaneous labour (reference), Emergency caesarean, Elective caesarean	Hospital admission with pneumonia and bronchitis
Green 2016^56^	England	Registry cohort	1970-89	1¶	243 708	Any caesarean	Hospital admission with bronchitis
Si 2022^61^	China	Cohort	2006-14	1.5-5	10 298	Vaginal spontaneous labour (reference), Caesarean on maternal request	Hospital admission with pneumonia
Kristensen 2015^34^	Denmark	Registry cohort	1997-2003	2	399 175	Emergency caesarean, Elective caesarean	Hospital admission with respiratory syncytial virus infection
Peters 2018^49^	Australia	Registry cohort	2000-13	5	491 590	Vaginal spontaneous labour (reference), Emergency caesarean with and without induction/augmentation, Elective caesarean	Hospital admission with respiratory and gastrointestinal infection
Betts 2021^50^	Australia	Registry cohort	2009-15	1	10 960	Spontaneous non-instrumental vaginal (reference), Non-medically indicated caesarean	Hospital admission with respiratory and gastrointestinal infections
Kristensen 2016^37^	Denmark	Registry cohort	1997-2012	14	790 569	Emergency caesarean, Elective caesarean	Hospital admission with laryngitis, lower respiratory, and gastrointestinal infection
Håkansson 2003^38^	Sweden	Registry cohort	1984-1997	1-13	863 846	Non-instrumental vaginal (reference), Any caesarean‡	Hospital admission with gastrointestinal infection
Bentley 2016^51^	Australia	Registry cohort	2001-12	6	893 360	Vaginal spontaneous labour (reference), Caesarean pre-labour, Spontaneous labour caesarean, Labour induced caesarean	Hospital admission with gastrointestinal infection
Fathima 2019^52^	Australia	Registry cohort	2000-14	15	367 476	Non-instrumental vaginal (reference), Emergency caesarean, Elective caesarean	Hospital admission with gastrointestinal infection
Barnes 2019^53^	Australia	Registry cohort	1996-2012	17	438 241	Instrumental vaginal (reference), Emergency caesarean, Elective caesarean	Hospital admission with skin infection
Hviid 2007^39^	Denmark	Registry cohort	1977-2001	15	1.5 million	Any caesarean	Hospital admission with viral meningitis
**Other infections**							
Merenstein 2011^44^	USA	Cohort	2006-07	3-6	522	Any caesarean	Infection episodes
Hyvönen 2023^40^	Finland	Cohort	2016-19	1	1052	Any caesarean	Infection episodes
Christensen 2018^35^§	Denmark	Cohort	2010-15	5	2431	Emergency caesarean, Elective caesarean	Hospital admission with infection and infections at home
Barnes 2019^54^	Australia	Registry cohort	2002-12	5	469 589	Emergency caesarean, Elective caesarean	Respiratory emergency department presentations
Keshet 2022^63^	Israel and UK	Registry cohort	1994-2019	5	401 431	Any caesarean‡	Primary care visits for respiratory infections
Magnus 2011^41^	Norway	Cohort	2001-10	3	37 171	Any caesarean‡	Recurrent lower respiratory tract infections
Langer 2022^60^	Germany	Cohort	2014-20	2	288	Any caesarean	Respiratory infection episodes
Hartley 2020^45^	Canada	Registry Cohort	2003-14	11	36 318	Any caesarean‡	Primary care visit or hospital admission for otitis media
Korvel-Hanquist 2018^42^	Denmark	Cohort	1996-2002	1.5	54 549	Any caesarean	Otitis media episodes
Bager 2010^43^	Denmark	Registry cohort	1973-2005	32	1.7 million	Any caesarean	Intestinal bacterial infections
Higgins 2021^57^	UK	Cohort	2010-16	1	412	Any caesarean	Infectious wheezing

* Follow-up age from birth up to the age listed unless a range is specified.

† Broadest infection outcome listed.

‡ Any caesarean and subgroups of emergency or elective caesarean.

§ Study listed twice in the table as outcomes included both infections in hospital and infections at home.

¶ Ongoing follow-up but 89% admissions <1 year.

### Risk of bias and quality of evidence assessment

Individual study assessments for risk of bias are shown in online supplemental figure 2 and confounder adjustments for each study are listed in online supplemental table 1. The overall risk of bias assessment was low for five studies,^48^,^5058 62^ some concerns for 15 studies,^2635 37 38 40 41 45^,^47 51 52 54 57 59 63^ high for eight studies,^34 36 39 42 43 53 55 56^ and very high for three studies.^44 60 61^ Most concerns related to the risk of bias from confounding, including no adjustment for factors that we considered important in our causal model or adjustment for mediators. We generally considered the risk of selection bias to be low because many of the studies were registry based and loss-to-follow-up in cohort studies was unlikely to be affected by exposure status due to the general nature of the included population-based cohorts. Information bias was assessed as minimal for the exposure and variable for the outcome but considered non-differential. Under the GRADE framework, the certainty of evidence was considered very low, but this largely reflects that all included studies were observational (online supplemental table 2).

### Hospital admission with infection

Twenty one studies analysed an outcome of hospital admission with infection. Of these studies, eight were included in meta-analyses.^2636^,^38 52 55 59 62^ Eight were excluded from meta-analyses because of overlapping populations,^3435 46^,^49 51 58^ three because of different exposure categorisations,^50 56 61^ and two because of dissimilar infection outcomes.^39 53^ The main findings for the studies excluded from meta-analyses are shown in online supplemental table 3.

**Figure 2:**
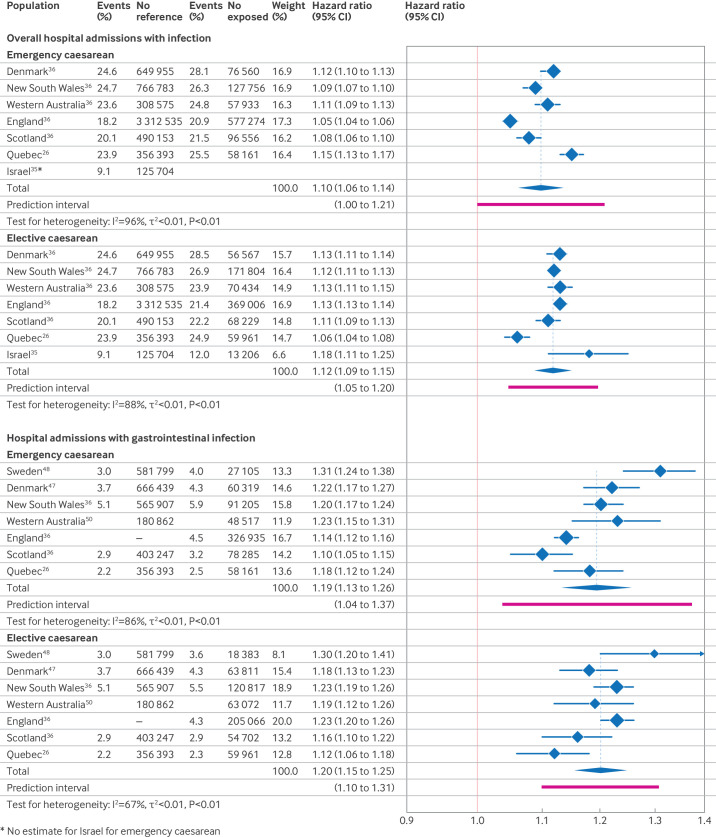
Forest plots showing the results of meta-analyses of the association between emergency and elective caesarean section and two infection outcomes related to hospital admission

Meta-analyses of overall hospital admission with infection included six study populations from two studies^26 62^ for emergency caesarean section and seven study populations from three studies^26 59 62^ for elective caesarean section. Compared with vaginally born children, individuals born by emergency caesarean section had an estimated 10% increased rate of hospital admission with infection (n=6, pooled HR 1.10 (95% confidence interval 1.06 to 1.14), τ^2^=0.0009, I^2^=96%) and individuals born by elective caesarean had a 12% increased rate (n=7, 1.12 (1.09 to 1.15), τ^2^=0.0006, I^2^=88%) (figure 2).

**Figure 3:**
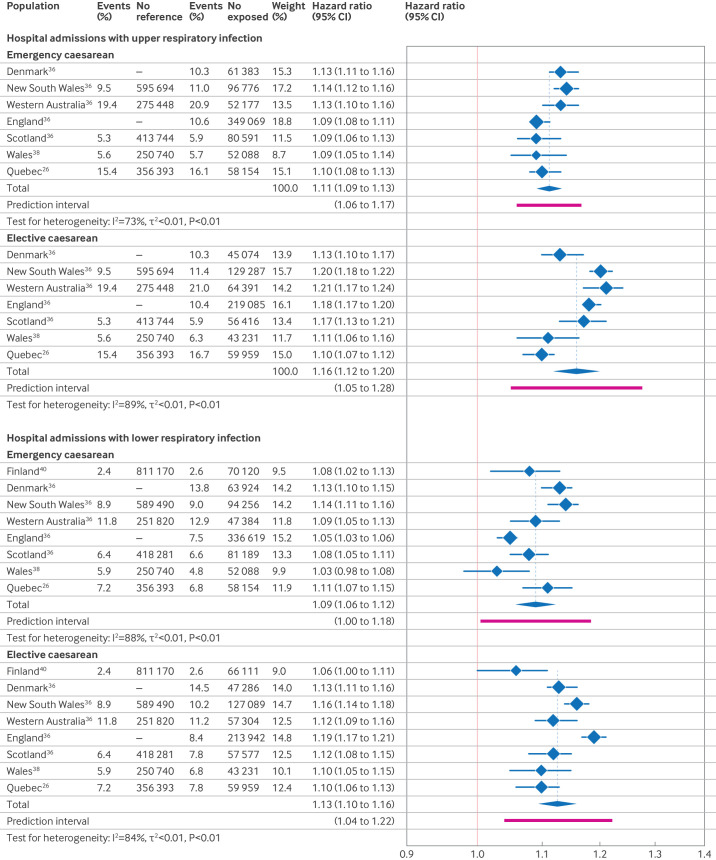
Forest plots showing the results of meta-analyses of the association between emergency and elective caesarean section and two infection outcomes related to hospital admission

Meta-analyses of hospital admission with upper respiratory infections included seven study populations from three studies^26 55 62^ and those of hospital admission with lower respiratory infections, included eight study populations from four studies.^26 36 55 62^ Children born by emergency caesarean section had an estimated 11% increased rate of these upper respiratory infections (n=7, pooled HR 1.11 (95% CI 1.09 to 1.13), τ^2^=0.0003, I^2^=73%) and a 9% increased rate of these lower respiratory infections (n=8, 1.09 (1.06 to 1.12), τ^2^=0.0010, I^2^=88%) compared with children born vaginally. Individuals born by elective caesarean had an estimated 16% increased rate of hospital admission with upper respiratory infections (n=7, 1.16 (1.12 to 1.20), τ^2^=0.0012, I^2^=89%) and a 13% increased rate of admission with lower respiratory infections (n=8, 1.13 (1.10 to 1.16), τ^2^=0.0009, I^2^=84%) compared with children born vaginally (figure 3). Sensitivity analyses to account for overlapping populations from Denmark used the estimate from Kristensen et al^37^ for lower respiratory infections in place of Miller et al^62^ and showed similar results (online supplemental figure 3).

Meta-analyses of hospital admission with gastrointestinal infections included seven study populations from five studies.^26 37 38 52 62^ Children born by emergency caesarean section had an estimated 19% increased rate of hospital admission with gastrointestinal infections (n=7, pooled HR 1.19 (1.13 to 1.26), τ^2^=0.0025, I^2^=86%) and children born by elective caesarean had a 20% increased rate (n=7, 1.20 (1.15 to 1.25), τ^2^=0.0009, I^2^=67%) compared with children born vaginally (figure 2). Sensitivity analyses, to account for overlapping populations from Denmark and Western Australia, used the estimates from Miller et al^62^ in place of Kristensen et al^37^ for Denmark and in place of Fathima et al^52^ for Western Australia and showed similar results (online supplemental figure 3).

The main findings for the studies which were excluded from meta-analyses are shown in online supplemental table 3. Collectively, the findings from these studies were consistent with those included in meta-analyses. Across these 13 studies, only one study for one type of caesarean section reported an effect estimate below the null; the confidence interval for this estimate was wide and included the null.^35^ Some studies examined instrumental vaginal birth separately to non-instrumental vaginal birth and included birth interventions such as induction of labour in mode of birth categorisations. These studies generally reported higher risk with instrumental vaginal and induced births (both vaginal and caesarean section), although with some exceptions.^47 48 53^ Studies were too few to explore follow-up age in our meta-analyses. In five studies,^26 42 43 51 62^ including subgroup analyses by age, a small attenuation with longer follow-up was noted, but this finding was not consistent nor particularly pronounced.

### Other infections

**Figure 4:**
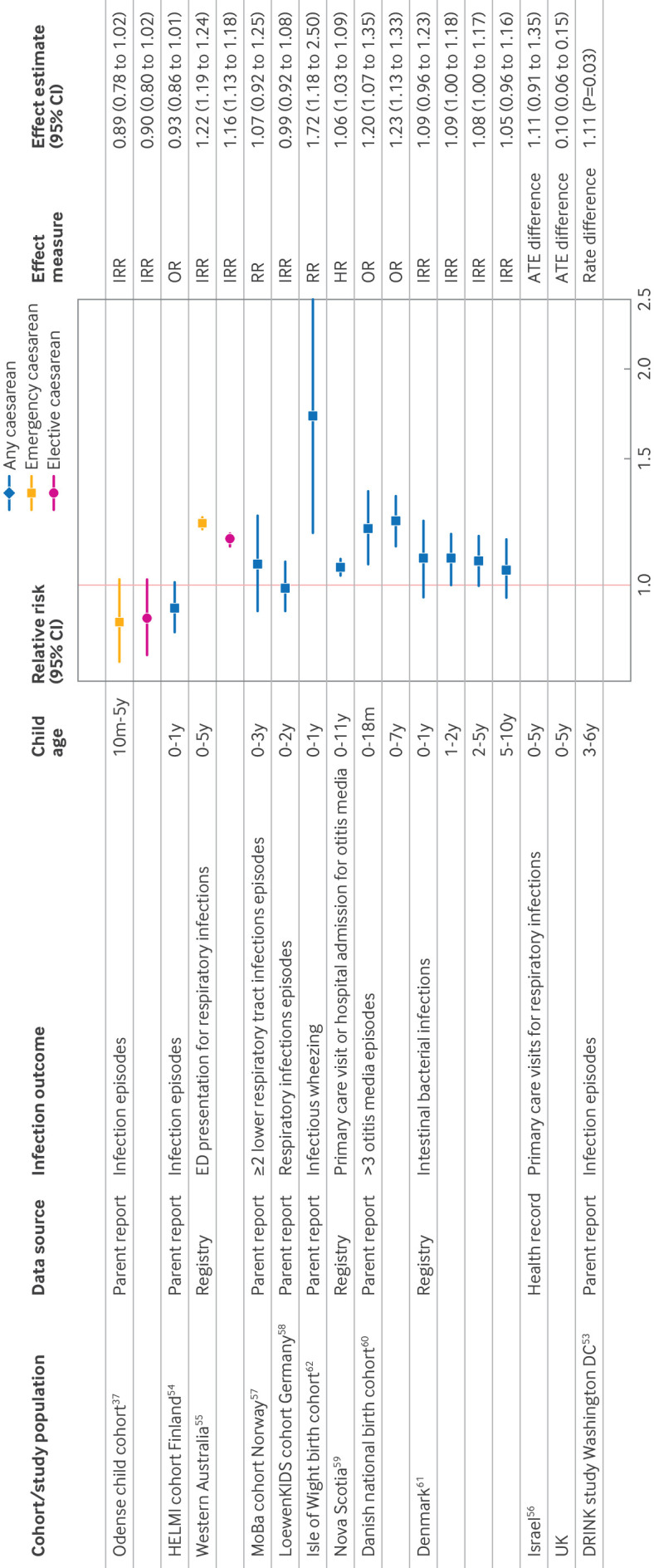
Forest plot of results from studies of other infections. Child age is indicated as months (m) or years (y). ATE=average treatment effect; HR=hazard ratio; IRR=incidence ratio; OR=odds ratio; RR=risk ratio

Eleven studies had a primary infection outcome that was not restricted to admission to hospital and largely reflected infections that did not lead to admission to hospital, including parent report of infection episodes, pathogen specific registries, primary care or emergency department visits for infections, and composite outcomes (eg, primary care and visits to hospital combined) (table 1). We did not combine these studies in a meta-analysis due to substantial variation in study design and infection outcomes. Figure 4 shows a forest plot of the main results from these studies. Eight of 11 of these studies suggested an increased risk of infection in caesarean born children compared with vaginally born children, although, several estimates included the null within their confidence interval (figure 4).

## Discussion

### Principal findings

This comprehensive systematic review and meta-analysis investigated the association between mode of birth and the risk of both general and specific types of infection in childhood. In 31 studies across 13 countries of over 10 million children, we report consistent findings of an association between caesarean section birth and increased risk of childhood infections either requiring hospital admission or not, beyond the neonatal period. Our meta-analyses indicated increased risks of admission to hospital related to overall, upper respiratory, lower respiratory, and gastrointestinal infection among children born by both emergency and elective caesarean section. Findings were consistent despite variation in setting, study design, and adjustment strategy.

### Comparison with similar research

A previous systematic review of respiratory tract infections, among other paediatric outcomes, reported an odds ratio of 1.30 (95% CI 1.06 to 1.60) based on three studies, but did not differentiate between infections of the upper and lower respiratory tract and had unclear methods on whether the crude or adjusted odds ratio was calculated.^14^ One of the studies included in this review pooled findings from five different populations and reported a pooled hazard ratio for overall hospital admissions with infection of 1.10 (95% CI 1.09 to 1.12),^62^ similar to the effect sizes in our study. No previous systematic reviews or meta-analyses of mode of birth and gastrointestinal infections, upper respiratory tract infections, or lower respiratory tract infections have been published.

### Strengths and limitations

This study is the largest and most comprehensive synthesis and meta-analysis of the literature regarding mode of birth and infections across childhood. We followed robust procedures for conducting a systematic review including pre-registration and adherence to a protocol, completing screening and quality assessment in duplicate, and reporting according to the PRISMA guidelines. We assessed the quality and risk of bias of each individual study.

Other strengths and weaknesses reflect those inherent to the studies included in the review. The studies had several methodological strengths. Firstly, mode of birth as the exposure is likely to be measured reliably because it was most often determined from the birth record, or in the case of self-report, parental recall was likely to be accurate. Similarly, most studies on hospital admission and infections used diagnoses from hospital registries coded using the ICD. However, some misclassification of diagnoses might have occurred because these data are primarily collected for administrative purposes rather than for research. The parameters used when assigning ICD-coded discharge diagnoses might vary between settings. Infection outcomes that did not lead to hospital admission might be subject to greater information bias due to self-report, but this would most likely be non-differential with respect to the exposure. We chose to exclude studies focused on vertically acquired and neonatal infections; however, some neonatal infections might have been included because some of the original studies did not discriminate between neonatal and post-neonatal infections. In our risk of bias assessment, we did not require adjustment for gestational age as one of our important confounders because it may be considered conceptually as a confounder or a mediator in different scenarios. However, all but four studies accounted for gestational age through statistical adjustment,^38 39 56 57^ restricting analyses to term pregnancies, or including sensitivity analyses. Many studies were registry based and captured entire populations, thereby reducing selection bias from both participation and loss-to-follow-up.

The study design and methodological quality varied between studies with heterogeneity in exposure categorisation, outcome definitions, follow-up age, confounder adjustments, and subgroup analyses. Overall, little diversity was reported; all the studies we identified were in populations from high income countries, reflecting the considerable infrastructure and resources necessary for population level data collection and large cohort studies. Several studies reported data for overlapping cohorts, which reduced the overall number of studies that could be included in meta-analyses. Furthermore, one study was particularly influential in its contribution to our pooled estimates from meta-analyses because this was a large, multinational, and high quality study.^62^ I^2^ is commonly used as a statistical measure of heterogeneity and was considered moderate to high in our meta-analyses. However, I^2^ is the proportion of the total variance that would remain if the variance due to sampling error is removed.^64^ Because our meta-analyses included studies with large sample sizes and therefore with corresponding high precision, the variance due to sampling error component will be small. Therefore, the high I^2^ values mainly reflect the precision of the included studies rather than large observable heterogeneity in the results. The calculated prediction intervals illustrate that although study estimates had some variation, the direction of effect was consistent. The certainty of evidence under the GRADE framework reflects that all the studies were observational; a randomised controlled trial of mode of birth would be unethical in most circumstances.

All studies attempted to control for confounding, but in many instances, the risk of bias assessment highlighted concerns regarding confounder selection, including data-driven confounder selection, adjustment for post-exposure variables, and lack of inclusion of confounding factors that we considered important based on our causal model. Despite concerns around inadequate and varying confounder adjustment across studies, the consistency of the findings was striking. This consistency may be indicative of a consistent effect across caesarean and infection categories or may also reflect residual confounding because all included studies were observational. Residual confounding could be through confounding by indication, where the reasons contributing to the decision to perform a caesarean section (eg, overweight or obesity, diabetes, hypertension, and medical conditions) or concurrent interventions (eg, intrapartum antibiotics and corticosteroid exposure to increase fetal lung maturity) may increase the risk of infection in offspring rather than the procedure itself.^2265^,^67^ Similarly, confounding from social patterning is possible whereby the differing social and consequent health characteristics of those who give birth by caesarean section versus vaginally may not be fully captured by adjusted covariates. One study did address confounding possibilities through a discordant sibling analysis with similar results to their overall population analysis.^58^ However, sibling analyses may still be biased through amplification of non-shared confounding factors between siblings.^68^

### Clinical and public health implications

More than a fifth of births occur by caesarean section^2^ and around a fifth of children are admitted to hospital with an infection by the age of 5 years.^62^ Therefore, even the modest increased risks we observed, ranging between 9-20% in meta-analyses, may represent an important health burden in terms of hospital admission and other health service usage.

Causality is difficult to infer from observational findings when the effect size is modest. While numerous studies have examined associations between mode of birth and childhood outcomes, researchers generally do not have information from birth records on why individual births occur by caesarean section—particularly regarding the medical indication, maternal preference, or both—only crude classifications of whether it is "elective/planned" or "emergency/acute". We note, for example, that elective caesarean section includes pregnancies complicated by pre-eclampsia, in addition to other complications such as placenta previa, which prohibit vaginal birth. This category of caesarean section births is therefore very heterogeneous and is not a marker of caesarean section conducted based on the woman's preference. Furthermore, these caesarean categories are likely to vary between settings making it difficult to interpret findings by type of caesarean section. Routine collection of more granular perinatal data that includes standardised indications for decisions on mode of birth and explicit definitions of mode of birth categories would assist in future research on possible long term associations with mode of birth. These data would allow more detailed examination of which factors are likely to be causal, the mechanisms through which they operate, and, therefore, how to intervene most effectively.

Further mechanistic studies will assist our understanding of the underlying biological pathways. In addition to indications and co-interventions of caesarean section births, many studies have pointed to the hypothesis that functional differences in the colonising microbiome in infants born by vaginal and caesarean section births may affect immune development and related outcomes.^69^ We postulate that elective caesarean sections are less exposed to maternal microflora as membranes are not ruptured, in contrast to those born by emergency caesarean, where rupture of membranes and exposure to labour could increase exposure to the maternal vaginal microbiome. However, we did not observe clear differences in our pooled estimates between emergency and elective caesarean section, possibly reflecting differences between settings in how types of caesarean section are categorised. Other mechanistic theories point to the potential impact on immune response of epigenetic alterations following caesarean section birth and associated intrapartum interventions.^22^ Mediation through early life factors may also partially explain the effect. For example, breastfeeding reduces the risk of childhood infection^70^ and breastfeeding initiation is generally lower following caesarean section birth.^71^ The findings of robust epidemiological observations may inform the design of mechanistic studies and intervention trials, which will provide the evidence to guide practice and policy.

In conclusion, our findings summarising results from high income countries show a consistent association between caesarean section birth and greater risk of infections in children. Limitations of existing studies include the potential for unmeasured confounding, specifically confounding by indication, and a lack of studies from low and middle income countries. Our epidemiological data may inform mechanistic studies to explore whether these associations are causal and, if so, development of safe, acceptable, and scalable interventions to reduce infection burden.

## Supplementary material

10.1136/bmjmed-2024-000995 online supplemental file 1

10.1136/bmjmed-2024-000995 online supplemental file 2

## Data Availability

Data are available upon reasonable request.
