# Supplementary material for: Caesarean section and risk of infection in offspring: systematic review and meta-analysis of observational studies
Source: BMJ Med. 2024 Nov 27;3(1):e000995. doi: 10.1136/bmjmed-2024-000995 (PMC11603743; doi:10.1136/bmjmed-2024-000995)
Supplement: online supplemental file 1 [file bmjmed-3-1-s001.pdf]

Supplementary material contents

Supplementary Figure 1 – Causal model

Supplementary Figure 2 - Risk of bias assessments for each study

Supplementary Table 1 – Confounder adjustments in individual studies

Supplementary Table 2 – Certainty of evidence and summary effect estimates assessed by GRADE

Supplementary Table 3 – Main findings from studies of hospitalised infection outcomes not included in meta-analyses

Supplementary Figure 3 - Sensitivity analyses for overlapping populations in meta-analyses.

Supplementary Appendix 1 – Review protocol and amendments

Supplementary Appendix 2 – PRISMA checklist

Supplementary Appendix 3 – Literature search strategy

Supplementary Figure 1 – Causal model

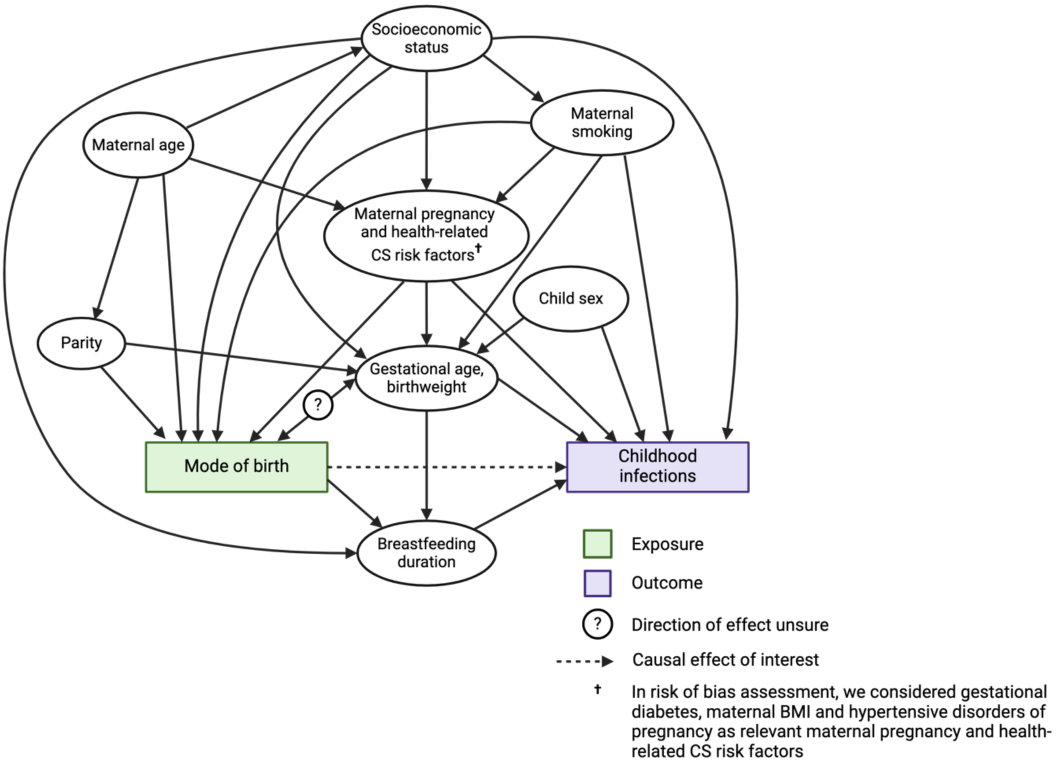

Figure created with BioRender.com

Supplementary Figure 2 - Risk of bias assessments for each study

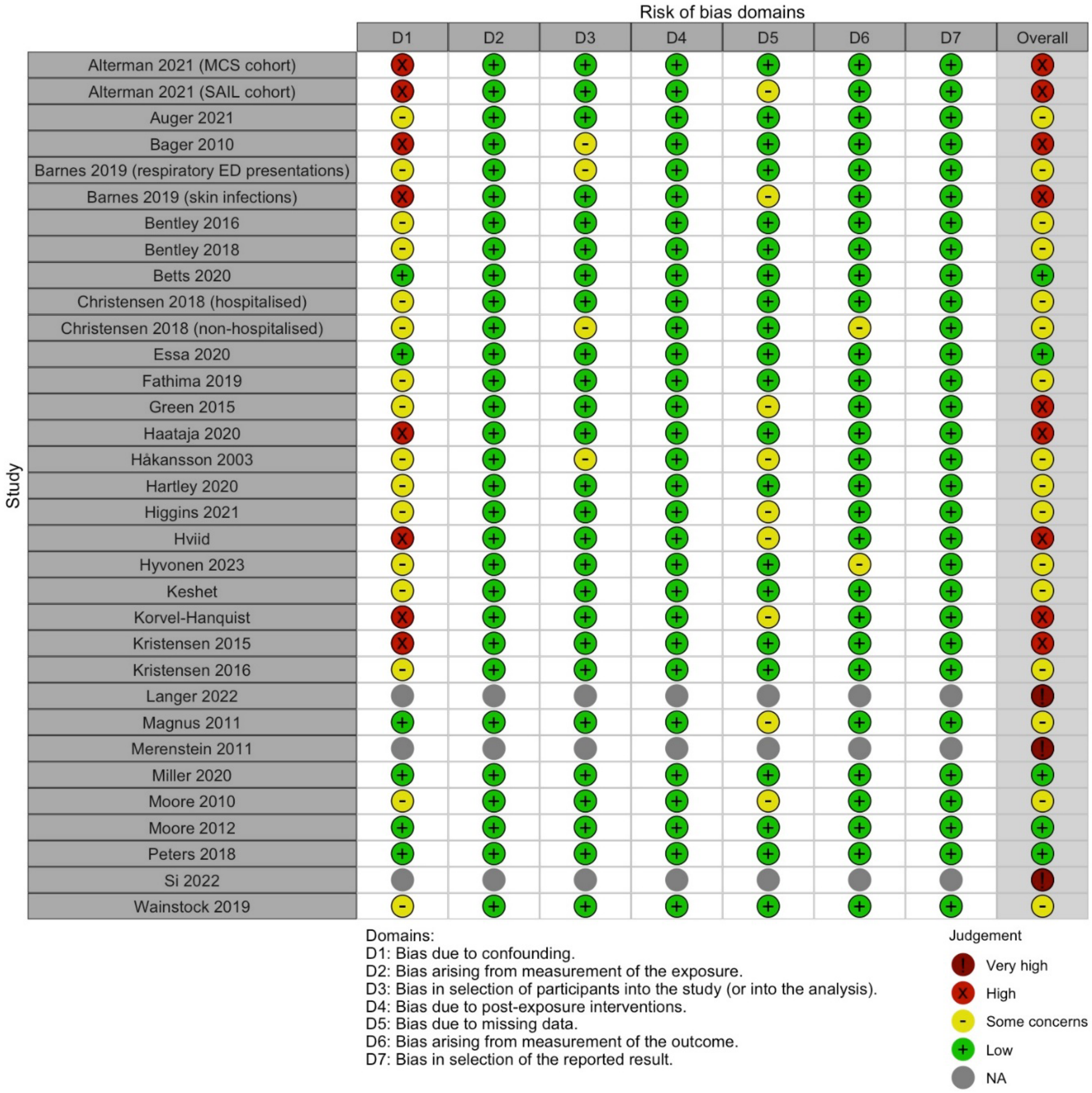

Figure created using the 'robvis' tool.[72]

Supplementary Table 1 - Confounder adjustments in each study

Reference	Covariates
**Hospitalised infections**	
Auger 2021[26]	Maternal age, parity, child sex, area-level SES, birth year, hypertensive disorders of pregnancy, maternal diabetes, illicit drug, tobacco and alcohol use, congenital anomalies, place of residence
Bentley 2018[33]	Maternal age, parity, child sex, birthweight, smoking during pregnancy, area-level SES, birth year, season of birth, maternal country of birth, marital status, diabetes during pregnancy, hypertension during pregnancy, neonatal length of stay z-score, infection during birth admission
Essa 2020[34]	Maternal age, child sex, birthweight, smoking during pregnancy, preeclampsia, maternal diabetes, gestational age <34 weeks, maternal obesity
Wainstock 2019[35]	Maternal age, child sex, birthweight, Apgar score, insufficient prenatal care, preterm birth
Miller 2020[36]	Maternal age, parity, gestational age, child sex, birthweight, smoking during pregnancy, area-level SES, birth year, season of birth, hypertensive disorders during pregnancy, maternal diabetes
Christensen 2018[37] [#]	Parity, gestational age, child sex, smoking during pregnancy, maternal BMI, maternal education level and for non-hospitalised infections additionally: age and season of year at response.
Alterman 2021[38]	Maternal age, gestational age, child sex, birthweight, area-level SES, birth year, season of birth, firstborn, maternal smoking, breastfeeding, maternal asthma, hypertensive conditions, ethnicity
Moore 2010[39]	Maternal age, parity, gestational age, child sex, smoking during pregnancy, area-level SES, birth year, season of birth, percent optimal birthweight, maternal asthma, remoteness index of Australia
Haataja 2020[40]	Maternal age, parity, gestational age, child sex, smoking during pregnancy, Apgar score, place of birth, region of birth, birthweight for gestational age, resuscitation at birth, ventilator support, antibiotic therapy in first week of life
Moore 2012[41]	Maternal age, parity, gestational age, child sex, smoking during pregnancy, area-level SES, birth year, season of birth, preeclampsia, gestational diabetes, breech presentation, maternal asthma, percent optimal birth weight
Green 2016[42]	Maternal age, parity, child sex, smoking during pregnancy, birth year, social class
Si 2022[43]	Maternal age, gestational age, child sex, birthweight, maternal BMI, maternal education level, maternal occupation, gestational weight gain, micronutrient supplementation during pregnancy, infant feeding, delivery hospital, medical insurance status
Kristensen 2015[44]	Parity, birthweight, smoking during pregnancy, prematurity, asphyxia, multiple births, single parenthood, asthma diagnoses up to 2 weeks before RSV hospitalisation
Peters 2018[45]	Maternal age, parity, gestational age, child sex, birthweight, area-level SES, maternal country of birth, pain medication during labour and birth, anaesthesia, small and large for gestational age, birth trauma
Betts 2021[46]	Propensity score weighting methods using several maternal and pregnancy characteristics. See published study for full details.
Kristensen 2016[47]	Maternal age, gestational age, child sex, birthweight, smoking during pregnancy, complications during pregnancy: preeclampsia, eclampsia, haemorrhage, hyperemesis.
Håkansson 2003[48]	Maternal age, child sex, birth year, smoking during pregnancy, maternal education level
Bentley 2016[49]	Maternal age, parity, gestational age, child sex, birthweight, smoking during pregnancy, area-level SES, breastfeeding initiation; maternal country of birth, hypertensive disorders of pregnancy, maternal diabetes, 5-minute Apgar score <7, infection during perinatal period, birth admission length of stay, birth pre/post July 2007
Fathima 2019[50]	Maternal age, parity, gestational age, child sex, smoking during pregnancy, area-level SES, season of birth, plurality, proportion of optimal birthweight, remoteness, birth pre/post May 2007
Barnes 2019[51]	Parity, gestational age, child sex, smoking during pregnancy, area-level SES, season of birth, plurality, percent optimal body weight, special care unit visit, remoteness, maternal diabetes
Hviid 2007[52]	None

Other infections	
Merenstein 2011[53]	Breastfeeding duration, child age, siblings, clustering within families (siblings enrolled in the study)
Hyvönen 2023[54]	Parity, child sex, birth year, intrapartum antibiotics, parental asthma, maternal education level, use of probiotics during pregnancy, furry pet in household
Christensen 2018[37] [#]	Parity, gestational age, child sex, smoking during pregnancy, maternal BMI, maternal education level and for non-hospitalised infections additionally: age and season of year at response.
Barnes 2019[55]	Maternal age, parity, gestational age, child sex, birthweight, smoking during pregnancy, area-level SES, season of birth, geographical region of residence, birthweight included with the percent optimal birth weight measure
Keshet 2022[56]	Propensity score weighting methods using several maternal and pregnancy characteristics. See published study for full details.
Magnus 2011[57]	Maternal age, parity, gestational age, child sex, birthweight, smoking during pregnancy, maternal BMI, breastfeeding duration, maternal marital status, maternal education level, maternal CS preference, previous CS delivery, chronic conditions before pregnancy, pregnancy complications, duration of membrane rupture, day care attendance at 18 months, maternal atopy
Langer 2022[58]	Parity, gestational age, child sex, breastfeeding duration, age commencing day-care
Hartley 2020[59]	Maternal age, parity, child sex, smoking during pregnancy, maternal weight, area-level SES, birthweight for gestational age, rural/urban residence
Korvel-Hanquist 2018[60]	Maternal age, parity, gestational age, child sex, birthweight, smoking during pregnancy, parental SES, maternal education, pre-pregnancy maternal smoking, daily contact to pets, following vaccination program, time before starting day care
Bager 2010[61]	Maternal age, parity, gestational age, child sex, birthweight, parental income, season of birth, age, degree of urbanisation, calendar year, county of residence
Higgins 2021[62]	Maternal age, smoking during pregnancy, maternal asthma, maternal infection, birth weight <2500g.

[#] Study listed twice in the table as outcomes included both hospitalised infections and infections at home

Supplementary Table 2 - Certainty of evidence and summary effect estimates assessed by GRADE (grading of recommendations, assessment, development, and evaluation) of the study outcomes.

Outcomes	Summary of findings				Quality assessment					Certainty of evidence
	No. of studies	No. of populations	Exposure category	Pooled HR (95% CI)	Study design[†]	Inconsistency[‡]	Indirectness[*]	Imprecision[§]	Other considerations[¶]	
Overall hospitalised infections	3	7	Elective CS	1.12 (1.09-1.15)	Not serious	Serious	Not serious	Not serious	NA	⊕OOO Very low
	2	6	Emergency CS	1.10 (1.06-1.14)						
Hospitalised upper respiratory infections	3	7	Elective CS	1.16 (1.12-1.20)	Not serious	Serious	Not serious	Not serious	NA	⊕OOO Very low
			Emergency CS	1.11 (1.09-1.13)						
Hospitalised lower respiratory infection	4	8	Elective CS	1.13 (1.10-1.16)	Not serious	Serious	Not serious	Not serious	NA	⊕OOO Very low
			Emergency CS	1.09 (1.06-1.12)						
Hospitalised gastrointestinal infections	5	7	Elective CS	1.20 (1.15-1.25)	Not serious	Serious	Not serious	Not serious	NA	⊕OOO Very low
			Emergency CS	1.19 (1.13-1.26)						
Other infections	11	12	Several	See comment[#]	Serious	Serious	Serious	Serious	NA	⊕OOO Very low

[#] Result not estimated in meta-analyses due to large differences in study design

[†] Downgraded one or two levels respectively if >25% or >50% of participants (meta-analysed outcomes) or included studies (not meta-analysed outcomes) are rated as high or very high risk of bias

[‡] Downgraded by one level if heterogeneity (I^2) >50%

[*] Downgraded one level if >25% of included studies were small cohorts (<5000 participants)

[§] Downgraded one level if the limits of the 95% confidence interval are wide or include values indicating both appreciable harm or benefit (risk estimates of ±10% or more)

[¶] Considered large magnitude of effect or if all residual confounding would decrease magnitude of effect (in situations with an effect). We did not consider publication bias as the number of studies was too small.

Supplementary Table 3 - Main findings from studies of hospitalised infection outcomes not included in meta-analyses

Study	Study population	Infection outcome	Child age	Effect measure	CS Type	Estimate (95% CI)
Bentley 2018	New South Wales	Overall hospitalised infections	0-5y	HR	Vaginal, spontaneous labour	Reference
					Vaginal, labour induction	1.13 (1.11-1.15)
					CS pre-labour	1.17 (1.15-1.19)
					CS spontaneous labour	1.13 (1.11-1.16)
					CS labour induction	1.21 (1.18-1.25)
Essa 2020	Southern region Israel	Overall hospitalised infections	0-18y	HR	Vaginal	Reference
					Any CS	1.25 (1.02-1.53)
Christensen 2018	Odense, Denmark	Overall hospitalised infections	0-5y	IRR	Vaginal	Reference
					Emergency CS	0.89 (0.68-1.18)
					Elective CS	1.45 (1.16-1.80)
Moore 2010	Western Australia	Hospitalised lower respiratory tract infections	0-2y	OR	Vaginal (instrumental)	Reference
					Vaginal (non-instrumental)	1.04 (0.96-1.13)
					Emergency CS	1.20 (1.09-1.33)
					Elective CS	1.34 (1.22-1.48)
Moore 2012	Western Australia	Hospitalised bronchiolitis	0-1y	IRR	Vaginal, spontaneous labour	Reference
					Vaginal (instrumental)	0.96 (0.85-1.08)
					Emergency CS	1.00 (0.89-1.13)
					Elective CS	1.11 (1.01-1.23)
		Hospitalised pneumonia	0-1y		Vaginal, spontaneous labour	Reference
					Vaginal (instrumental)	
					Emergency CS	
					Elective CS	1.03 (0.80-1.33)
Green 2016	South East England	Hospitalised bronchiolitis		OR	Vaginal	Reference
					Any CS	1.35 (1.03-1.53)
Si 2022	Hebei Province, China	Hospitalised pneumonia	1.5-5y		Vaginal, spontaneous labour	Reference
					CS on maternal request	1.16 (0.94-1.45)
Kristensen 2015	Denmark	Hospitalised RSV infection	0-2y	HR	Vaginal	Reference
					Emergency CS	1.09 (1.01-1.17)
					Elective CS	1.27 (1.19-1.36)

Study	Study population	Infection outcome	Child age	Effect measure	CS Type	Estimate (95% CI)
Peters 2018	New South Wales	Hospitalised respiratory infections	0-5y	OR	Vaginal, spontaneous labour	Reference
					Vaginal with induction/augmentation	1.11 (1.08-1.13)
					Instrumental vaginal without induction/augmentation	1.25 (1.20-1.31)
					Instrumental vaginal with induction/augmentation	1.31 (1.27-1.36)
					Emergency CS without induction/augmentation	1.39 (1.32-1.46)
					Emergency CS after induction/augmentation	1.29 (1.23-1.34)
					Elective CS	1.35 (1.31-1.40)
		Hospitalised gastrointestinal infections	0-5y		Vaginal (spontaneous)	Reference
					Vaginal with induction/augmentation	1.22 (1.09-1.37)
					Instrumental vaginal without induction/augmentation	1.13 (0.90-1.41)
					Instrumental vaginal with induction/augmentation	0.96 (0.79-1.16)
					Emergency CS without induction/augmentation	1.24 (0.98-1.57)
					Emergency CS after induction/augmentation	1.19 (0.98-1.45)
					Elective CS	1.21 (1.02-1.44)
Betts 2021	Queensland	Hospitalised respiratory infections	0-1y	OR	Vaginal birth	Reference
					Non-medically indicated CS	1.52 (0.99-2.31)
		Hospitalised gastrointestinal infections	0-1y		Vaginal birth	Reference
					Non-medically indicated CS	2.21 (1.25-3.89)
Bentley 2016	New South Wales	Hospitalised gastrointestinal infections	0-6y	HR	Vaginal, spontaneous labour	Reference
					Vaginal, labour induction	1.12 (1.09-1.15)
					CS pre-labour	1.19 (1.16-1.23)
					CS spontaneous labour	1.20 (1.16-1.25)
					CS labour induction	1.23 (1.18-1.29)
Barnes 2019	Western Australia	Hospitalised skin infection	0-17y	HR	Vaginal (instrumental)	Reference
					Vaginal (non-instrumental)	1.07 (0.97-1.17)
					Emergency CS	1.10 (0.98-1.22)
					Elective CS	1.09 (0.97-1.23)
Hviid 2007	Denmark	Hospitalised viral meningitis	0-15y	IRR	Vaginal	Reference
					Any CS	1.29 (1.12-1.49)

Supplementary Figure 3 – Sensitivity analyses for overlapping populations in meta-analyses.

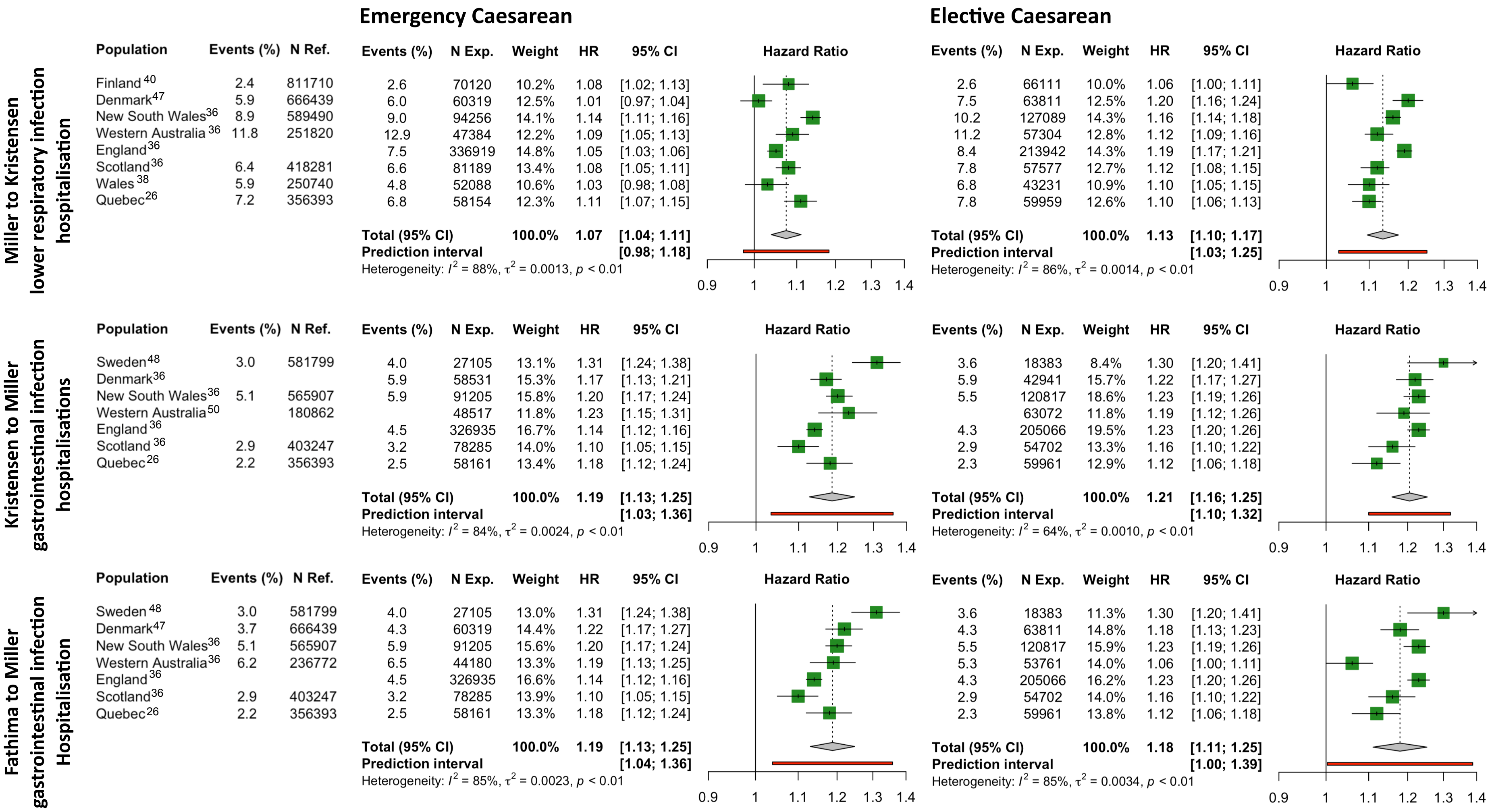

Miller to Kristensen lower respiratory infection hospitalisation

Population	Events (%)	N Ref.	Emergency Caesarean Events (%)	N Exp.	Weight	HR	95% CI	Elective Caesarean Events (%)	N Exp.	Weight	HR	95% CI
Finland[40]	2.4	811710	2.6	70120	10.2%	1.08	[1.02; 1.13]	2.6	66111	10.0%	1.06	[1.00; 1.11]
Denmark[47]	5.9	666439	6.0	60319	12.5%	1.01	[0.97; 1.04]	7.5	63811	12.5%	1.20	[1.16; 1.24]
New South Wales[36]	8.9	589490	9.0	94256	14.1%	1.14	[1.11; 1.16]	10.2	127089	14.3%	1.16	[1.14; 1.18]
Western Australia[36]	11.8	251820	12.9	47384	12.2%	1.09	[1.05; 1.13]	11.2	57304	12.8%	1.12	[1.09; 1.16]
England[36]			7.5	336919	14.8%	1.05	[1.03; 1.06]	8.4	213942	14.3%	1.19	[1.17; 1.21]
Scotland[36]	6.4	418281	6.6	81189	13.4%	1.08	[1.05; 1.11]	7.8	57577	12.7%	1.12	[1.08; 1.15]
Wales[38]	5.9	250740	4.8	52088	10.6%	1.03	[0.98; 1.08]	6.8	43231	10.9%	1.10	[1.05; 1.15]
Quebec[26]	7.2	356393	6.8	58154	12.3%	1.11	[1.07; 1.15]	7.8	59959	12.6%	1.10	[1.06; 1.13]
**Total (95% CI)**					**100.0%**	**1.07**	**[1.04; 1.11]**			**100.0%**	**1.13**	**[1.10; 1.17]**
**Prediction interval**							**[0.98; 1.18]**					**[1.03; 1.25]**

Emergency Caesarean – Heterogeneity: I^2 = 88%, τ^2 = 0.0013, p < 0.01
Elective Caesarean – Heterogeneity: I^2 = 86%, τ^2 = 0.0014, p < 0.01

Kristensen to Miller gastrointestinal infection hospitalisations

Population	Events (%)	N Ref.	Emergency Caesarean Events (%)	N Exp.	Weight	HR	95% CI	Elective Caesarean Events (%)	N Exp.	Weight	HR	95% CI
Sweden[48]	3.0	581799	4.0	27105	13.1%	1.31	[1.24; 1.38]	3.6	18383	8.4%	1.30	[1.20; 1.41]
Denmark[36]			5.9	58531	15.3%	1.17	[1.13; 1.21]	5.9	42941	15.7%	1.22	[1.17; 1.27]
New South Wales[36]	5.1	565907	5.9	91205	15.8%	1.20	[1.17; 1.24]	5.5	120817	18.6%	1.23	[1.19; 1.26]
Western Australia[50]		180862		48517	11.8%	1.23	[1.15; 1.31]		63072	11.8%	1.19	[1.12; 1.26]
England[36]			4.5	326935	16.7%	1.14	[1.12; 1.16]	4.3	205066	19.5%	1.23	[1.20; 1.26]
Scotland[36]	2.9	403247	3.2	78285	14.0%	1.10	[1.05; 1.15]	2.9	54702	13.3%	1.16	[1.10; 1.22]
Quebec[26]	2.2	356393	2.5	58161	13.4%	1.18	[1.12; 1.24]	2.3	59961	12.9%	1.12	[1.06; 1.18]
**Total (95% CI)**					**100.0%**	**1.19**	**[1.13; 1.25]**			**100.0%**	**1.21**	**[1.16; 1.25]**
**Prediction interval**							**[1.03; 1.36]**					**[1.10; 1.32]**

Emergency Caesarean – Heterogeneity: I^2 = 84%, τ^2 = 0.0024, p < 0.01
Elective Caesarean – Heterogeneity: I^2 = 64%, τ^2 = 0.0010, p < 0.01

Fathima to Miller gastrointestinal infection Hospitalisation

Population	Events (%)	N Ref.	Emergency Caesarean Events (%)	N Exp.	Weight	HR	95% CI	Elective Caesarean Events (%)	N Exp.	Weight	HR	95% CI
Sweden[48]	3.0	581799	4.0	27105	13.0%	1.31	[1.24; 1.38]	3.6	18383	11.3%	1.30	[1.20; 1.41]
Denmark[47]	3.7	666439	4.3	60319	14.4%	1.22	[1.17; 1.27]	4.3	63811	14.8%	1.18	[1.13; 1.23]
New South Wales[36]	5.1	565907	5.9	91205	15.6%	1.20	[1.17; 1.24]	5.5	120817	15.9%	1.23	[1.19; 1.26]
Western Australia[36]	6.2	236772	6.5	44180	13.3%	1.19	[1.13; 1.25]	5.3	53761	14.0%	1.06	[1.00; 1.11]
England[36]			4.5	326935	16.6%	1.14	[1.12; 1.16]	4.3	205066	16.2%	1.23	[1.20; 1.26]
Scotland[36]	2.9	403247	3.2	78285	13.9%	1.10	[1.05; 1.15]	2.9	54702	14.0%	1.16	[1.10; 1.22]
Quebec[26]	2.2	356393	2.5	58161	13.3%	1.18	[1.12; 1.24]	2.3	59961	13.8%	1.12	[1.06; 1.18]
**Total (95% CI)**					**100.0%**	**1.19**	**[1.13; 1.25]**			**100.0%**	**1.18**	**[1.11; 1.25]**
**Prediction interval**							**[1.04; 1.36]**					**[1.00; 1.39]**

Emergency Caesarean – Heterogeneity: I^2 = 85%, τ^2 = 0.0023, p < 0.01
Elective Caesarean – Heterogeneity: I^2 = 85%, τ^2 = 0.0034, p < 0.01

Supplementary Appendix 1

Review protocol

Caesarean section versus vaginal mode of birth and risk of infectious morbidity in the offspring: a systematic review and meta-analysis of observational studies

To enable PROSPERO to focus on COVID-19 submissions, this registration record has undergone basic automated checks for eligibility and is published exactly as submitted. PROSPERO has never provided peer review, and usual checking by the PROSPERO team does not endorse content. Therefore, automatically published records should be treated as any other PROSPERO registration. Further detail is provided here.

Citation

Isobel Todd, Jessica Miller, David Burgner, Lars Henning Pedersen, Maria Magnus. Caesarean section versus vaginal mode of birth and risk of infectious morbidity in the offspring: a systematic review and meta-analysis of observational studies. PROSPERO 2022 CRD42022369252 Available from:
https://www.crd.york.ac.uk/prospero/display_record.php?ID=CRD42022369252

Review question

What is the effect of caesarean section compared to vaginal mode of birth on risk of hospitalised and non-hospitalised infections in children up to 18 years of age?

Searches

We will search the electronic databases MEDLINE, EMBASE and PubMed. No restrictions on language, dates, and locations of the studies will be applied.

Types of study to be included

Inclusion criteria:

1. Original observational studies using a case-control, cohort (follow-up or registry-based) or cross-sectional design.

Exclusion criteria:

1. Studies based on non-population-based sample (that is, the study population is selected for a specific condition or characteristic of interest rather than recruitment from the general population)

2. Studies focused on vertically-acquired infections.

Condition or domain being studied

Mode of birth (caesarean section and vaginal). Infections in children.

Participants/population

Children less than 18 years of age.

Intervention(s), exposure(s)

Caesarean section (also known as c-section or caesarean birth/delivery).

Comparator(s)/control

Vaginal birth/delivery.

Context

Studies focused on general populations. That is, the study sample/recruitment methods should be such that they are emulating (or attempting to emulate) a random sample from a general population.

Main outcome(s)

Childhood infections. This will include both non-hospitalised infections (e.g. self-reported or doctor-diagnosed) and hospitalised infections. Hospitalised infections are typically defined by ICD diagnosis associated with the hospital record, although the codes used to define 'infection-related' can vary. We will examine hospitalised infections overall from studies that report effect measures for general/overall hospitalisations due to infection, as per their definitions. For types of hospitalised infection and for non-hospitalised infections we will be guided by the results of the search as to the appropriate grouping of infection outcomes. Reported results will either be grouped by: (1) clinical types of infection (e.g., respiratory, gastrointestinal, invasive bacterial), (2) specific pathogens (e.g., RSV, influenza) or, (3) specific syndromes (e.g., bronchiolitis, pneumonia, otitis media).

Measures of effect

Measure of effect will be any relative measure of association (e.g. hazard/risk/rate/odds ratio) comparing caesarean with vaginal birth.

Additional outcome(s)

Not applicable

Data extraction (selection and coding)

Study selection:

Results of the search strategy will be imported into Covidence. Two reviewers (IT and JM) will independently screen the titles and abstracts of the database searches for studies that meet the selection criteria and are relevant for full text retrieval with a PRISMA chart to record the screening and selection process.

Full text screening:

Full text screening will then be performed by IT and JM to identify studies that will be included in the systematic review and meta-analysis. A third reviewer (DB) will be consulted to resolve any disagreements.

Data extraction:

Data to be extracted from eligible papers include the names of authors, title of paper, year of publication, country/region of origin, study design, study objectives, participant characteristics, how exposures and outcomes were defined, adjusted covariates, measure of association for primary and secondary outcomes.

IT will extract the data into an excel spreadsheet and JM will check the extracted data. Authors of eligible studies will be contacted by email for missing data required for the systematic review and meta-analysis.

Risk of bias (quality) assessment

Cochrane risk of bias tools will be used to assess risk of bias in different study designs (e.g. RoB 2, ROBINS-I)

Strategy for data synthesis

Narrative synthesis:

The narrative synthesis will be presented in both text and tabular formats. A table of the study findings and quality assessment of the studies will be created. The narrative synthesis will implement the Synthesis Without Meta-analysis (SWiM) reporting guideline.

Meta-analysis:

The exposure and outcome will be assessed for suitability for data pooling to perform a meta-analysis. Data pooling may be possible for only some of the main and secondary outcomes. The I^2 statistic will be used to assess heterogeneity between studies with values of 25%, 50%, and 75% used to indicate low, moderate, and high heterogeneity, respectively. Publication bias will be assessed using forest and funnel plots.

Analysis of subgroups or subsets

We will perform the following subgroup analyses if sufficient data are available: age at follow up (e.g. neonatal (birth-1m), infant (1m-1y), pre-school (2-4y), school-age (5-9y), adolescent (10y+)), type of infection (as outlined above in main outcomes), child sex, type of caesarean section (acute/emergency versus planned/elective), and country income level (low-and-middle income versus high income).

Contact details for further information

Isobel Todd

isobel.todd@mcri.edu.au

Organisational affiliation of the review

Murdoch Children's Research Institute

https://www.mcri.edu.au/

Review team members and their organisational affiliations

Ms Isobel Todd. Murdoch Children's Research Institute

Dr Jessica Miller. Murdoch Children's Research Institute

Professor David Burgner. Murdoch Children's Research Institute

Professor Lars Henning Pedersen. Aarhus University

Dr Maria Magnus. Norwegian Institute of Public Health

Type and method of review

Epidemiologic, Meta-analysis, Narrative synthesis, Systematic review

Anticipated or actual start date

31 October 2022

Anticipated completion date

31 March 2023

Funding sources/sponsors

Ms Todd's PhD is supported by an Australian Government Research Training Program (RTP) Scholarship.

Conflicts of interest

Language

English

Country

Australia, Denmark, Norway

Stage of review

Review Ongoing

Subject index terms status

Subject indexing assigned by CRD

Subject index terms

Humans

Date of registration in PROSPERO

12 November 2022

Date of first submission

01 November 2022

Stage of review at time of this submission

**Stage**	**Started**	**Completed**
Preliminary searches	Yes	No
Piloting of the study selection process	No	No
Formal screening of search results against eligibility criteria	No	No
Data extraction	No	No
Risk of bias (quality) assessment	No	No
Data analysis	No	No

The record owner confirms that the information they have supplied for this submission is accurate and complete and they understand that deliberate provision of inaccurate information or omission of data may be construed as scientific misconduct.

The record owner confirms that they will update the status of the review when it is completed and will add publication details in due course.

Versions

12 November 2022

12 November 2022

Protocol amendments

The types of study to be included was updated from our initial registered protocol. Changes are shown below in red:

Types of study to be included

Inclusion criteria:
1. Original observational studies using a case-control or cohort (follow-up or registry-based) or cross-sectional design.

Exclusion criteria:

1. Studies based on non-population-based sample (that is, the study population is selected for a specific condition or characteristic of interest rather than recruitment from the general population)

2. Studies focused on vertically-acquired or neonatal infections.

3. Studies where the outcome was prevalent rather than incident cases of infection

4. Studies which have not undergone peer review

Supplementary Appendix 2

PRISMA checklist

PRISMA 2020 Checklist

Section and Topic	Item #	Checklist item	Location where item is reported
**TITLE**			
Title	1	Identify the report as a systematic review.	Page 1
**ABSTRACT**			
Abstract	2	See the PRISMA 2020 for Abstracts checklist.	
**INTRODUCTION**			
Rationale	3	Describe the rationale for the review in the context of existing knowledge.	Page 4
Objectives	4	Provide an explicit statement of the objective(s) or question(s) the review addresses.	Page 4
**METHODS**			
Eligibility criteria	5	Specify the inclusion and exclusion criteria for the review and how studies were grouped for the syntheses.	Page 5-6
Information sources	6	Specify all databases, registers, websites, organisations, reference lists and other sources searched or consulted to identify studies. Specify the date when each source was last searched or consulted.	Page 5
Search strategy	7	Present the full search strategies for all databases, registers and websites, including any filters and limits used.	Supplementary Appendix 3
Selection process	8	Specify the methods used to decide whether a study met the inclusion criteria of the review, including how many reviewers screened each record and each report retrieved, whether they worked independently, and if applicable, details of automation tools used in the process.	Page 5-6
Data collection process	9	Specify the methods used to collect data from reports, including how many reviewers collected data from each report, whether they worked independently, any processes for obtaining or confirming data from study investigators, and if applicable, details of automation tools used in the process.	Page 6
Data items	10a	List and define all outcomes for which data were sought. Specify whether all results that were compatible with each outcome domain in each study were sought (e.g. for all measures, time points, analyses), and if not, the methods used to decide which results to collect.	Page 5
	10b	List and define all other variables for which data were sought (e.g. participant and intervention characteristics, funding sources). Describe any assumptions made about any missing or unclear information.	Page 6
Study risk of bias assessment	11	Specify the methods used to assess risk of bias in the included studies, including details of the tool(s) used, how many reviewers assessed each study and whether they worked independently, and if applicable, details of automation tools used in the process.	Page 6
Effect measures	12	Specify for each outcome the effect measure(s) (e.g. risk ratio, mean difference) used in the synthesis or presentation of results.	Page 6
Synthesis methods	13a	Describe the processes used to decide which studies were eligible for each synthesis (e.g. tabulating the study intervention characteristics and comparing against the planned groups for each synthesis (item #5)).	Page 6
	13b	Describe any methods required to prepare the data for presentation or synthesis, such as handling of missing summary statistics, or data conversions.	Page 6
	13c	Describe any methods used to tabulate or visually display results of individual studies and syntheses.	Page 6
	13d	Describe any methods used to synthesize results and provide a rationale for the choice(s). If meta-analysis was performed, describe the model(s), method(s) to identify the presence and extent of statistical heterogeneity, and software package(s) used.	Page 6-7
	13e	Describe any methods used to explore possible causes of heterogeneity among study results (e.g. subgroup analysis, meta-regression).	Page 6[#]
	13f	Describe any sensitivity analyses conducted to assess robustness of the synthesized results.	Page 6
Reporting bias assessment	14	Describe any methods used to assess risk of bias due to missing results in a synthesis (arising from reporting biases).	Page 7*
Certainty assessment	15	Describe any methods used to assess certainty (or confidence) in the body of evidence for an outcome.	Page 6

PRISMA 2020 Checklist

Section and Topic	Item #	Checklist item	Location where item is reported
assessment			
**RESULTS**			
Study selection	16a	Describe the results of the search and selection process, from the number of records identified in the search to the number of studies included in the review, ideally using a flow diagram.	Figure 1
	16b	Cite studies that might appear to meet the inclusion criteria, but which were excluded, and explain why they were excluded.	Page 10
Study characteristics	17	Cite each included study and present its characteristics.	Table 1, Supplementary Table 1
Risk of bias in studies	18	Present assessments of risk of bias for each included study.	Supplementary Figure 2
Results of individual studies	19	For all outcomes, present, for each study: (a) summary statistics for each group (where appropriate) and (b) an effect estimate and its precision (e.g. confidence/credible interval), ideally using structured tables or plots.	Figure 2, Figure 3, Supplementary Table 3
Results of syntheses	20a	For each synthesis, briefly summarise the characteristics and risk of bias among contributing studies.	Page 10, Supplementary Figure 2
	20b	Present results of all statistical syntheses conducted. If meta-analysis was done, present for each the summary estimate and its precision (e.g. confidence/credible interval) and measures of statistical heterogeneity. If comparing groups, describe the direction of the effect.	Page 10-11, Figure 2 & 3
	20c	Present results of all investigations of possible causes of heterogeneity among study results.	Page 11[#]
	20d	Present results of all sensitivity analyses conducted to assess the robustness of the synthesized results.	Page 10-11, Supplementary Figure 3
Reporting biases	21	Present assessments of risk of bias due to missing results (arising from reporting biases) for each synthesis assessed.	Page 7*
Certainty of evidence	22	Present assessments of certainty (or confidence) in the body of evidence for each outcome assessed.	Page 10, Supplementary Table 2
**DISCUSSION**			
Discussion	23a	Provide a general interpretation of the results in the context of other evidence.	Page 12
	23b	Discuss any limitations of the evidence included in the review.	Page 12-13
	23c	Discuss any limitations of the review processes used.	Page 13
	23d	Discuss implications of the results for practice, policy, and future research.	Page 13-14
**OTHER INFORMATION**			
Registration and protocol	24a	Provide registration information for the review, including register name and registration number, or state that the review was not registered.	Page 5
	24b	Indicate where the review protocol can be accessed, or state that a protocol was not prepared.	Page 5
	24c	Describe and explain any amendments to information provided at registration or in the protocol.	Supplementary Appendix 1

PRISMA 2020 Checklist

Section and Topic	Item #	Checklist item	Location where item is reported
Support	25	Describe sources of financial or non-financial support for the review, and the role of the funders or sponsors in the review.	Funding statement
Competing interests	26	Declare any competing interests of review authors.	Competing interests statement
Availability of data, code and other materials	27	Report which of the following are publicly available and where they can be found: template data collection forms; data extracted from included studies; data used for all analyses; analytic code; any other materials used in the review.	Data access statement

*As noted on Page 7, the number of studies was too few to explore publication/reporting bias
#The number of studies included in meta-analyses was too small to perform further sub-group analyses or meta-regression

From: Page MJ, McKenzie JE, Bossuyt PM, Boutron I, Hoffmann TC, Mulrow CD, et al. The PRISMA 2020 statement: an updated guideline for reporting systematic reviews. BMJ 2021;372:n71. doi: 10.1136/bmj.n71
For more information, visit: http://www.prisma-statement.org/

PRISMA 2020 extension for abstracts

Section and Topic	Item #	Checklist item	Reported (Yes/No)
**TITLE**			
Title	1	Identify the report as a systematic review.	Yes
**BACKGROUND**			
Objectives	2	Provide an explicit statement of the main objective(s) or question(s) the review addresses.	Yes
**METHODS**			
Eligibility criteria	3	Specify the inclusion and exclusion criteria for the review.	Yes
Information sources	4	Specify the information sources (e.g. databases, registers) used to identify studies and the date when each was last searched.	Yes
Risk of bias	5	Specify the methods used to assess risk of bias in the included studies.	Yes
Synthesis of results	6	Specify the methods used to present and synthesise results.	Yes
**RESULTS**			
Included studies	7	Give the total number of included studies and participants and summarise relevant characteristics of studies.	Yes
Synthesis of results	8	Present results for main outcomes, preferably indicating the number of included studies and participants for each. If meta-analysis was done, report the summary estimate and confidence/credible interval. If comparing groups, indicate the direction of the effect (i.e. which group is favoured).	Yes
**DISCUSSION**			
Limitations of evidence	9	Provide a brief summary of the limitations of the evidence included in the review (e.g. study risk of bias, inconsistency and imprecision).	Yes
Interpretation	10	Provide a general interpretation of the results and important implications.	Yes
**OTHER**			
Funding	11	Specify the primary source of funding for the review.	Yes
Registration	12	Provide the register name and registration number.	Yes

Supplementary Appendix 3

Literature search strategy

EMBASE

1. cesarean section/
2. vaginal birth after cesarean/
3. (mode-of-birth or mode-of-delivery or delivery-mode or birth-mode or method-of-delivery or method-of-birth or caesarean or cesarean or cesarian or caesarian or cesarien or caesarien or c-section or vaginal-birth*).tw,kf,dq.
4. 1 or 2 or 3
5. (newborn* or new-born* or baby or babies or neonat* or neo-nat* or infan* or toddler* or pre-schooler* or preschooler* or kinder or kinders or kindergarten* or kinder-aged or boy or boys or girl or girls or child or children or childhood or pediatric* or paediatric* or school-age* or schoolage* or schoolchild* or schoolgirl* or schoolboy* or adolescen* or youth or youths or teen or teens or teenage*).af.
6. exp infection/
7. exp communicable disease/
8. (infection* or infectious).tw,kf,dq.
9. 6 or 7 or 8
10. (risk or risks).tw,kf,hw,dq.
11. 4 and 5 and 9 and 10
12. observational study/
13. cohort analysis/
14. longitudinal study/
15. follow up/
16. retrospective study/
17. exp case control study/
18. cross-sectional study/
19. quasi experimental study/
20. prospective study/
21. (observational adj3 (study or studies or design or analysis or analyses)).ti,ab,kf.
22. cohort*.ti,ab,kf.
23. (prospective adj7 (study or studies or design or analysis or analyses)).ti,ab,kf.
24. ((follow up or followup) adj7 (study or studies or design or analysis or analyses)).ti,ab,kf.
25. ((longitudinal or longterm or (long adj term)) adj7 (study or studies or design or analysis or analyses or data)).ti,ab,kf.
26. (retrospective adj7 (study or studies or design or analysis or analyses or data or review)).ti,ab,kf.
27. ((case adj control) or (case adj comparison) or (case adj controlled)).ti,ab,kf.
28. (case-referent adj3 (study or studies or design or analysis or analyses)).ti,ab,kf.
29. (population adj3 (study or studies or analysis or analyses)).ti,ab,kf.
30. (descriptive adj3 (study or studies or design or analysis or analyses)).ti,ab,kf.
31. ((multidimensional or (multi adj dimensional)) adj3 (study or studies or design or analysis or analyses)).ti,ab,kf.
32. (cross adj sectional adj7 (study or studies or design or research or analysis or analyses or survey or findings)).ti,ab,kf.
33. ((natural adj experiment) or (natural adj experiments)).ti,ab,kf.
34. (quasi adj (experiment or experiments or experimental)).ti,ab,kf.
35. ((non experiment or nonexperiment or non experimental or nonexperimental) adj3 (study or studies or design or analysis or analyses)).ti,ab,kf.
36. (prevalence adj3 (study or studies or analysis or analyses)).ti,ab,kf.
37. case series.ti,ab,kf.
38. 12 or 13 or 14 or 15 or 16 or 17 or 18 or 19 or 20 or 21 or 22 or 23 or 24 or 25 or 26 or 27 or 28 or 29 or 30 or 31 or 32 or 33 or 34 or 35 or 36 or 37
39. 11 and 38
40. limit 39 to (conference abstract or conference paper or "conference review" or editorial or letter)
41. 39 not 40

MEDLINE

1. *cesarean section/ or *vaginal birth after cesarean/
2. (mode-of-birth or mode-of-delivery or delivery-mode or birth-mode or method-of-delivery or method-of-birth or caesarean or cesarean or cesarian or caesarian or cesarien or caesarien or c-section or vaginal-birth*).tw,kf.
3. 1 or 2
4. (newborn* or new-born* or baby or babies or neonat* or neo-nat* or infan* or toddler* or pre-schooler* or preschooler* or kinder or kinders or kindergarten* or kinder-aged or boy or boys or girl or girls or child or children or childhood or pediatric* or paediatric* or school-age* or schoolage* or schoolchild* or schoolgirl* or schoolboy* or adolescen* or youth or youths or teen or teens or teenage*).af.
5. exp *Infections/co, di, ep [Complications, Diagnosis, Epidemiology]
6. exp *Communicable Diseases/co, di, ep [Complications, Diagnosis, Epidemiology]
7. (infection* or infectious).tw,kf.
8. 5 or 6 or 7
9. (risk or risks).tw,kf,hw.
10. 3 and 4 and 8 and 9
11. Epidemiologic Methods/
12. exp Epidemiologic Studies/
13. Observational Studies as Topic/
14. Clinical Studies as Topic/
15. single-case studies as topic/
16. (Observational Study or Validation Studies or Clinical Study).pt.
17. (observational adj3 (study or studies or design or analysis or analyses)).ti,ab,kf.
18. cohort*.ti,ab,kf.
19. (prospective adj7 (study or studies or design or analysis or analyses)).ti,ab,kf.
20. ((follow up or followup) adj7 (study or studies or design or analysis or analyses)).ti,ab,kf.
21. ((longitudinal or longterm or (long adj term)) adj7 (study or studies or design or analysis or analyses or data)).ti,ab,kf.
22. (retrospective adj7 (study or studies or design or analysis or analyses or data or review)).ti,ab,kf.
23. ((case adj control) or (case adj comparison) or (case adj controlled)).ti,ab,kf.
24. (case-referent adj3 (study or studies or design or analysis or analyses)).ti,ab,kf.
25. (population adj3 (study or studies or analysis or analyses)).ti,ab,kf.
26. (descriptive adj3 (study or studies or design or analysis or analyses)).ti,ab,kf.
27. ((multidimensional or (multi adj dimensional)) adj3 (study or studies or design or analysis or analyses)).ti,ab,kf.
28. (cross adj sectional adj7 (study or studies or design or research or analysis or analyses or survey or findings)).ti,ab,kf.
29. ((natural adj experiment) or (natural adj experiments)).ti,ab,kf.
30. (quasi adj (experiment or experiments or experimental)).ti,ab,kf.
31. ((non experiment or nonexperiment or non experimental or nonexperimental) adj3 (study or studies or design or analysis or analyses)).ti,ab,kf.
32. (prevalence adj3 (study or studies or analysis or analyses)).ti,ab,kf.
33. case series.ti,ab,kf.
34. 11 or 12 or 13 or 14 or 15 or 16 or 17 or 18 or 19 or 20 or 21 or 22 or 23 or 24 or 25 or 26 or 27 or 28 or 29 or 30 or 31 or 32 or 33
35. 10 and 34
36. limit 35 to (case reports or comment or editorial or guideline or letter or practice guideline)
37. 35 not 36

PUBMED

Title/abstract
#1 “mode-of-birth” OR “mode-of-delivery” OR “delivery-mode” OR “birth-mode” OR “method-of-delivery” OR “method-of-birth” OR “caesarean” OR “cesarean” OR “cesarian” OR “caesarian” OR “cesarien” OR “caesarien” OR “c-section” OR “vaginal-birth*”

Title/abstract
#2 “newborn*” OR “new-born*” OR “baby” OR “babies” OR “neonat*” OR “neo-nat*” OR “infan*” OR “toddler*” OR “pre-schooler*” OR “preschooler*” OR “kinder” OR “kinders” OR “kindergarten*” OR “kinder-aged” OR “boy” OR “boys” OR “girl” OR “girls” OR “child” OR “children” OR “childhood” OR “pediatric*” OR “paediatric*” OR “school-age*” OR “schoolage*” OR “schoolchild*” OR “schoolgirl*” OR “schoolboy*” OR “adolescen*” OR “youth” OR “youths” OR “teen” OR “teens” OR “teenage*”

Title/abstract
#3 “infection*” OR “infectious” OR “communicable-disease*”

Title/abstract
#4 “risk” OR “risks”

#5 NOTNLM OR publisher[sb] OR inprocess[sb] OR pubmednotmedline[sb] OR indatareview[sb] OR pubstatusaheadofprint

#6 #1 AND #2 AND #3 AND #4 AND #5

Title/Abstract
#7 epidemiolog* AND (study OR studies OR design OR analysis OR analyses)

Title/Abstract
#8 Clinical-stud*

Title/Abstract
#9 observational AND (study OR studies OR design OR analysis OR analyses)

Title/Abstract
#10 cohort*

Title/Abstract
#11 prospective AND (study OR studies OR design OR analysis OR analyses)

Title/Abstract
#12 (follow-up OR followup) AND (study OR studies OR design OR analysis OR analyses)

Title/Abstract
#13 (longitudinal OR longterm OR long-term) AND (study OR studies OR design OR analysis OR analyses OR data)

Title/Abstract
#14 retrospective AND (study OR studies OR design OR analysis OR analyses OR data OR review)

Title/Abstract
#15 case-control OR case-comparison OR case-controlled

Title/Abstract
#16 case-referent AND (study OR studies OR design OR analysis OR analyses)

Title/Abstract
#17 population AND (study OR studies OR analysis OR analyses)

Title/Abstract
#18 descriptive AND (study OR studies OR design OR analysis OR analyses)

Title/Abstract
#19 (multidimensional OR multi-dimensional) AND (study OR studies OR design OR analysis OR analyses)

Title/Abstract
#20 Cross-sectional AND (study OR studies OR design OR research OR analysis OR analyses OR survey OR findings)

Title/Abstract
#21 natural-experiment OR natural-experiments

Title/Abstract
#22 Quasi-experiment*

Title/Abstract
#23 ("non experiment" OR nonexperiment OR "non experimental" OR nonexperimental) AND (study OR studies OR design OR analysis OR analyses)

Title/Abstract
#24 prevalence AND (study OR studies OR analysis OR analyses)

Title/Abstract
#25 case series

#26 #7 OR #8 OR #9 OR #10 OR #11 OR #12 OR #13 OR #14 OR #15 OR #16 OR #17 OR #18 OR #19 OR #20 OR #21 OR #22 OR #23 OR #24 OR #25

#27 #6 AND #26

Excluded books and document; case reports; comments; editorial; guideline; letter; practice guidelines
