# Supplementary material for: Caesarean section and risk of infection in offspring: systematic review and meta-analysis of observational studies
Source: BMJ Med. 2024 Nov 27;3(1):e000995. doi: 10.1136/bmjmed-2024-000995 (PMC11603743; doi:10.1136/bmjmed-2024-000995)
Supplement: Supplementary file 3 [file bmjmed-2024-000995.reviewer_comments.pdf]

PEER REVIEW HISTORY

BMJ Medicine publishes all reviews undertaken for accepted manuscripts. Reviewers are asked to complete a checklist review form and are provided with free text boxes to elaborate on their assessment. These free text comments are reproduced below.

This paper was submitted to a another journal from BMJ but declined for publication following peer review. The authors addressed the reviewers' comments and submitted the revised paper to BMJ Medicine. The paper was subsequently accepted for publication at BMJ Medicine.

ARTICLE DETAILS

**TITLE (PROVISIONAL)**	Caesarean section and risk of infection in offspring: a systematic review and meta-analysis of observational studies
**AUTHORS**	Todd, Isobel; Magnus, Maria Christine; Pedersen, Lars; Burgner, David; Miller, Jessica

VERSION 1 - REVIEW

**REVIEWER NAME**	Reviewer 1
**REVIEWER AFFILIATION**	
**REVIEWER CONFLICT OF INTEREST**	None
**DATE REVIEW RETURNED**	28-Apr-2024

**GENERAL COMMENTS**	This is a meta-analysis of studies looking at infection rates among children born by cesarean. The paper was well written and convincing. I have no comments on the methodology which looked clear and appropriate.<br><br>I have only one major comment – please address the mechanisms by which cesarean could lead to childhood infections. Is it the microbiome? Some other pathway? Greater attention to the pathways in the introduction and discussion would help.<br><br>Minor comment: Ambulatory might be a better term than non-hospitalized?

**REVIEWER NAME**	Reviewer 2
**REVIEWER AFFILIATION**	
**REVIEWER CONFLICT OF INTEREST**	None
**DATE REVIEW RETURNED**	28-Apr-2024

**GENERAL COMMENTS**	This study explores the associations between caesarean section and risk of infection in offspring, using a systematic review and meta -analysis of observational studies. This topic is innovative and the research content is interesting, but the method and results section needed to be improved, as commented below:<br><br>Major comments:<br>1. The description for infection outcomes is a little confusing. Should the infection outcomes be firstly classified into three categories, and

	they were also categorized by specificity of infection, as mentioned in method section (p5, line 31-37)? If so, ① The description in data synthesis and analysis was inconsistent (p6, line 34-36). ② Dose "hospitalized overall infections" incorporated "hospitalized upper and lower respiratory infections" and "hospitalized gastrointestinal infection"? if so, studies ref 35, 44, 45, 47 should be included in the meta-analysis of hospitalized overall infections. ③In results section, "Hospitalized overall infections", "Hospitalized upper and lower respiratory infections" and "Hospitalized gastrointestinal infections" are part of "Hospitalized infections", and they are not parallel to other parts of results section; therefore, removing these three subtitles might be more logical. ④Page 5, line31; page 10, table 1; page 12, line 25: What is the relationship between "non-hospitalized infection" and "other infection". Are they equal to each other?<br>2. For studies not included in meta-analysis but in systematic review, their results were needed to be mentioned in main text not only in tables or figures.<br>3. P5, line 37: how to distinguish prevalent and incident cases, especially for those upper respiratory infections.<br>4. heterogeneity test was lacking in this study. It should be included in methods and results section. If high heterogeneity was found, then additional analyses such as subgroup analysis or meta-regression could be performed.<br>5. In introduction section, the necessity of conducting this meta-analysis (no synthesis of the research, p4, line 20) seems not sufficient.<br><br><br>Minor comment：<br>6. In method section (P5), it might be more rational to put study search forward of study exclusion criteria.<br>7. Page 6 line 22-24: why the four factors were selected as important confounders. Maternal BMI might be more important than maternal smoking during pregnancy.<br>8. Page 13, line 41: please spell the full name of "ICD" when it was firstly presented.<br>9. In discussion section, the OR 1.3 was different from this study. The reason underlying this difference is needed to be mentioned.

**REVIEWER NAME**	Reviewer 3
**REVIEWER AFFILIATION**	
**REVIEWER CONFLICT OF INTEREST**	None
**DATE REVIEW RETURNED**	28-Apr-2024

**GENERAL COMMENTS**	Review BMJ: Todd et al. CS and risk of infection in offspring: a systematic review and meta analysis.<br><br>This is a very thoroughly conducted systematic review and meta-analysis of observational studies about a very important topic: the association between CS and childrens' infections with/without required hospital admissions.<br>The literature search is up to date and also all relevant/important papers have been included in the search. The research team has conducted the systematic review very well, by firstly publishing their study protocol in Prospero and besides the main findings also including very relevant supplementary materials regarding bias (s1) and results of studies which were not included in the meta analysis

	but which main findings are also relevant regarding the research topic (S2). Very well done, it was a pleasure to review this paper. Regarding each section, I have the following comments:<br><br>1. ABSTRACT<br>1.1 Data sources: the phrase inception is a little odd, please add the specific period of time to inform the reader.<br><br>2. INTRODUCTION<br>2.1 Though the introduction is very short, it contains all the important background and objectives. I would stress some more that conducting this research by including observational studies is highly relevant since it is unethical to perform an RCT regarding this matter. Moreover, based on other RCT's which are more related to onset of labour, 70-90% of women decline participation and therefore this systematic review and meta-analyses are of great importance particularly due to the increase of CS in Western high income countries.<br>2.2 Could you elaborate a little more about reasons for women to get an elective or emergency CS? So the reader is more informed about the differences in the provision of either elCS or emCS.<br>2.3 In the paragraph it is stated "examining different levels of infection severity and clinical infections categories", these phrases do not further appear in the manuscript. Please make this consistent to prevent confusion.<br>2.4 The references can be also updated by adding some of the studies of Supplementary materials 2. Or are the references stated here from systematic reviews/meta-analyses only? Please make this more consistent or clear.<br><br>3. METHODS<br>The inclusion of studies regarding observational studies by including case control or cohort (follow-up or registry based) and excluding non-population based studies is very well written and clearly described.<br>3.1 MAJOR. My comment regarding the project is related to the modes of births in the study. The modes of births of interest are elective CS and emergency CS, these will be separately compared with vaginal births. These vaginal births are grouped with spontaneous or instrumental (forceps or vacuum extraction) vaginal births. Based on our own previous work we observed that compared with spontaneous vaginal births, also children born with instrumental vaginal births had higher odds of infections (respiratory, gastrointestinal and other infections, Peters et al. 2018). So I was wondering why did you not decide to conduct the analyses with all modes of births separately:<br>-Spontaneous vaginal birth (=reference)<br>-Instrumental vaginal birth<br>-Elective CS<br>-Emergency CS<br>Moreover, why did you not mention the results (ORs) of instrumental vaginal births in the supplementary table 2?<br>3.2 MAJOR. This comment is related to the included population of interest. Did you include studies reporting results of term pregnancies only (≥ 37 weeks)? Or did you also include studies in the analyses with (very) pre term birth for which adjustments were made in the multivariable regression model? And if so: was gestational age an important confounder and therefore a requirement for inclusion? Otherwise, my concern will be that the (very) pre-term birth is highly associated with children's infections

	and not the mode of birth.<br>3.3 The categorization of the outcomes (i.e. infections) is quite detailed with four different categories. However, throughout the manuscript there is no mentioning on these categories since the infection categories are broadly described (hospital versus non-hospital/other infections). Please make this consistent throughout the manuscript to enhance clarity.<br>3.4 In the statistical paragraph you state: “Due to overlapping study populations across studies, we considered several study features to determine which results to statistically synthesise in meta-analyses according to the following hierarchy (….) (iii) similarity of the effect measure type (e.g., odds, hazard, or risk ratio) as the odds ratio is not an appropriate estimate of the risk ratio for non-rare outcomes” What is the difference between the odds and the odds ratio? Do you mean something differently? As an epidemiologist I do agree that reporting the HR is more preferred than the OR. However, can you back this statement up with a reference as well?<br>3.5 MAJOR. Concerning the outcome and the time that an infection can occur, is it very relevant to have such long-term follow-up period of the children? Particularly the studies reporting HRs up till 18 years of age. Many other factors may have contributed to particular infections? In the study protocol in Prospero there is mentioned that subgroup analyses (e.g. age, gender, country income level) will also be performed, did you perform a subgroup analysis for age-groups?<br>3.6 The studies reporting ORs in this field of research are also very relevant and important information, which you also show in supplementary table S2. This is highly appreciated since you show results for the HRs and ORs regarding the study aim. Thank you for being so thoroughly.<br><br>4. RESULTS<br>The results are very objectively and clearly written.<br>4.1 Regarding Table 1, I would adapt the title by informing the reader from the beginning if the mentioned studies included hospital infections an non hospital infections. Also, in the table the main exposure of interest (i.e. modes of birth) is reported, I observed that only the study of Barnes 2019 included instrumental vaginal birth? Is this also the reason why you have grouped them together (see also previous comment)?<br>4.2 In table 1 different sample sizes are included from studies with small (n=288) and large groups (=> 1 million). Are these different sample sizes influencing your results? Do you have to take this in to account in the statistical analysis?<br>4.3 MAJOR. Regarding the manuscript, hospitalised infections. In this paragraph it is mentioned why some studies are in/excluded in the meta analyses. The phrase overlapping populations is triggering me since are these populations really overlapping or did they select different pregnancies e.g. all pregnancies versus healthy pregnancies? Moreover, maybe some studies were also excluded because these reported ORS (Peters et al) and therefore results were shown in Supplementary Tables? Also there is more information shown in Figure 1 Flowchart, please make this more consistent since this is really relevant to know as a reader.<br>4.4 Regarding Figure 1 (flowchart)<br>Please add an ‘exclusion box’ between 5398 title and abstracts screened and 124 full text assessed for eligibility.<br>Please consider to add two additional boxes under 31 studies included for:<br>Hospital infections (including number of studies) and non-hospitalised infections (including number of studies) and could you

	add the range of period of time as well for the included studies?<br><br>5. DISCUSSION<br>5.1 MAJOR. The discussion includes all relevant sections (strengths, limitations, recommendations etc). I really appreciate the paragraph about the confounder by indication, since this is a major concern which has been very well addressed by the authors. It would suggest to elaborate some more about the total trajectory of labour and birth regarding other factors that are contributing to increased risk of infections (e.g. induction of labour, gestational age, and long-term follow-up period).<br>5.2 MAJOR. It would be valuable to have some reflection on the registration of infections across countries. For example, infections are very well registered in Australia and perhaps therefore there is a very high prevalence on infections in secondary care. Furthermore, there might be data classification issues regarding the classification on elective CS and emergency CS in electronic health registry data. These data are primarily for routine care and secondary for research which may cause some misclassification.<br>5.2 MAJOR. The reflection on results (comparison similar research) is based on OR's instead of HRs. Why did you not compare your results with HR, particularly since the OR's mentioned are part of the supplementary materials. Moreover, you made quite a statement in the statistical paragraph (methods) about your preference for HR.<br>5.3 The phrase about causality (page 14, sentence 33-34) is not correct since it is impossible to infer causality from the results of observational studies.<br>5.4 I would reflect some more about the bias in the included studies, particularly since you have created a very nice overview presented in the supplementary materials.<br>5.5 It would be very worthwhile to reflect some more about the values of the HRs for clinical practice. What could be the practical implication for this in daily care?<br>5.6 The impact of CS on infections is also reflected with the microbiome however it is lacking the potential interaction with epigenetics effects as well. Please see this paper and consider to add this as well from Dahlen et al 2016: Childbirth and consequent atopic disease: emerging evidence on epigenetic effects based on the hygiene and EPIIC hypotheses - PubMed (nih.gov)<br>5.7 Finally, your last paragraph you state: across a variety of settings? What do you mean by this? Several high income countries? A variety of settings, opens a whole new box for me, for example I think about several maternity care settings. Which is in my opinion not what you want to phrase here, please be scientifically objective as you have perfectly done throughout the manuscript.

**REVIEWER NAME**	Reviewer 4
**REVIEWER AFFILIATION**	
**REVIEWER CONFLICT OF INTEREST**	None
**DATE REVIEW RETURNED**	28-Apr-2024

**GENERAL COMMENTS**	The present systematic review and meta-analysis was performed to synthesize the evidence on the risk of hospitalised and non-hospitalised infections in children born by C-section compared to children born by vaginal birth. The review is well planned and conducted and the manuscript is very well written. The authors may

	wish to consider the following comments:<br><br>In the review protocol, the authors planned to include cohort, case-control or cross-sectional studies. In the eligibility criteria section of the manuscript, they only listed cohort or case-control studies. Although it is understandable that cross-sectional studies may not be eligible if they were done to estimate the prevalence rather than an association, some retrospective cohort studies, or even longitudinal cohort studies are sometimes described as cross-sectional studies. Cross-sectional studies are included in the search strategy, which is the right thing to do but the authors should clarify this point in this section as to how they handled this issue.<br><br>The authors searched PubMed and Embase but they should have searched Web of Science to ensure a comprehensive search.<br><br>Did IT and JM do the data extraction independently? I assume two review authors did the data extraction but this should be stated.<br><br>The authors pre-specified the key potential confounders in the risk of bias assessment. They listed maternal age, smoking, socio-economic status and "a maternal risk factor for caesarean section". Do they mean just one risk factor for C-section? Which factor is this? Or do they mean any risk factor for C-section? There is a long list of risk factors for C-section and the authors should be clear here.<br><br>Does ROBINS-E provide an overall score or another formula that enables researchers to classify study into low, moderate or high risk of bias?<br><br>Considering that all eligible studies are observational, what was the highest score that can be allocated to each study? Were all studies downgraded because they are observational, rather than RCTs, or all studies started at the highest grade? Some detail about the factors that led to downgrading would be helpful. Also, most of the tables and figures in the appendix are presented in a way that is hard for reviewing on a computer.<br><br>On page 14, line 8, the authors state "a randomised controlled trial of mode of birth is clearly unethical". Although I understand the authors' rationale for making this statement, I am not sure if there were evidence to back it up. It is understood that randomising women to have a C-section without medical indication is contentious, but saying that it is unethical should be backed by evidence.<br><br>Considering the small magnitude of the reported estimates, and the potential unmeasured confounding, the authors should take these issues into account in their conclusions. Previous studies on C-section and child morbidity found that some of the reported associations are related to unmeasured familial confounding rather than causal associations. This is important considering that the authors argue that these results "should inform the design of mechanistic studies and intervention trials…". If the observed associations are not causal they may end up misleading the design of future studies, especially intervention trials. Also, the authors suggest that breastfeeding may partially explain the effect of C-section on the risk of infections in the child. Shouldn't breastfeeding be considered a potential mediator rather than a confounder and therefore it is not explain the association in terms of causality but

	rather mediate the association?

VERSION 1 – AUTHOR RESPONSE

Reviewer 1
This is a meta-analysis of studies looking at infection rates among children born by cesarean. The paper was well written and convincing. I have no comments on the methodology which looked clear and appropriate.
I have only one major comment – please address the mechanisms by which cesarean could lead to childhood infections. Is it the microbiome? Some other pathway? Greater attention to the pathways in the introduction and discussion would help.
Thank you for this comment. We have revised the following sentence in the introduction as follows: "*We considered sub-types of caesarean delivery (e.g., emergency and elective) due to varying hypotheses on the biological mechanisms, particularly related to acquisition of the maternal microbiome, through which mode of birth may influence oMspring health21 22 and included both overall and specific clinical infection categories.*"
We have also revised the following section in the discussion section to include more detail about the potential pathways: *"We postulate that elective caesarean sections are less exposed to maternal microflora as membranes are not ruptured, in contrast to those born by emergency CS, where rupture of membranes and exposure to labour could increase exposure to the maternal vaginal microbiome. However, we did not observe clear diMerences in our pooled estimates between emergency and elective caesarean section possibly reflecting diMerences between settings in how types of caesarean section are categorised. Other mechanistic theories point to the potential impact on immune response of epigenetic alterations following caesarean section birth and associated intrapartum interventions.22 Mediation through early life factors may also partially explain the eMect. For example, breastfeeding reduces the risk of childhood infection67 and breastfeeding initiation is generally lower following caesarean section birth."*
Minor comment: Ambulatory might be a better term than non-hospitalized?
We suspect that this term is something that varies by setting and agree that it is diFicult to find the best term. Non-hospitalised is a more familiar term to us, however we have included ambulatory in our first description of non-hospitalised in response to your comment.

Reviewer 2
Comments:
This study explores the associations between caesarean section and risk of infection in oFspring, using a systematic review and meta-analysis of observational studies. This topic is innovative and the research content is interesting, but the method and results section needed to be improved, as commented below:
Major comments:
1. The description for infection outcomes is a little confusing. Should the infection outcomes be firstly classified into three categories, and they were also categorized by specificity of infection, as mentioned in method section (p5, line 31-37)? If so, ① The description in data synthesis and analysis was inconsistent (p6, line 34-36). ② Dose "hospitalized overall infections" incorporated "hospitalized upper and lower respiratory infections" and "hospitalized gastrointestinal infection"? if so, studies ref 35, 44, 45, 47 should be included in the meta-analysis of hospitalized overall infections. ③In results section, "Hospitalized overall infections", "Hospitalized upper and lower respiratory infections" and "Hospitalized gastrointestinal infections" are part of "Hospitalized infections", and they are not parallel to other parts of results section; therefore, removing these three subtitles might be more logical. ④Page 5, line31; page 10, table 1; page 12, line 25: What is the relationship between "non-hospitalized infection" and "other infection". Are they equal to each other?
Thank you for this comment. We have gone through each section and revised the text to provide better clarity as to the classification of infections. In answer to the question regarding hospitalised infections - the meta-analysis outcome 'hospitalised overall infections' is a synthesis of studies where the outcome was a measure of all infectionrelated hospitalisations combined. This includes upper and lower respiratory and

gastrointestinal infections among other clinical categories of infections. The other three meta-analyses (upper respiratory, lower respiratory and gastrointestinal infections) synthesise studies where the outcome is this specific clinical category of infection. We have revised the following sentence in the methods section to address this: *"As a result of this hierarchy, we were able to perform meta-analyses using the reported hazard ratios for elective/planned caesarean section and emergency/acute caesarean section separately for four hospitalised infection outcomes: overall hospitalised infections and three common clinical infection categories (upper respiratory, lower respiratory and gastrointestinal infections)."*

With respect to non-hospitalised infections, the terms 'other' and 'non-hospitalised' infections are used synonymously here. An inherent limitation that we attempted to address was that some studies had 'parent reported infections' as the outcome and these may have been either non-hospitalised or hospitalised, hence our use of the term 'other' infections. However these studies will largely represent non-hospitalised infections, which are much more common. We hope the additional changes to the wording throughout the manuscript improves the clarity regarding these issues. In particular, we have revised the results section *"Eleven studies had a primary infection outcome that was not restricted to hospitalisation and largely reflect non-hospitalised infections including parent-report of infection episodes, pathogen-specific registries and primary care or emergency department visits for infections, and composite outcomes (e.g., primary care and hospitalisation visits combined) (**Table 1**)."*

2. For studies not included in meta-analysis but in systematic review, their results were needed to be mentioned in main text not only in tables or figures.

Thank you for this comment. We have revised the results section to include an extra paragraph summarising the results presented in supplementary table 2 as follows: *"The main findings for the studies which were excluded from meta-analyses are shown in **Supplementary Table 2**. Collectively, the findings from these studies were consistent with those included in meta-analyses. Across these 13 studies, only one study for one type of caesarean section reported an eMect estimate below the null and the confidence interval for this estimate was wide and included the null.34 Some studies examined instrumental vaginal birth separately to non-instrumental vaginal birth and included birth interventions such as induction of labour in mode of birth categorisations. These studies generally reported higher risk with instrumental vaginal and induced births (both vaginal and caesarean section), albeit with some exceptions.36,38,48 There were too few studies to explore follow-up age in our meta-analyses. In five studies26,33,46,57,58 including sub-group analyses by age, there was a small attenuation with longer follow-up, but this was not consistent nor particularly pronounced."*

3. P5, line 37: how to distinguish prevalent and incident cases, especially for those upper respiratory infections.

By prevalent cases we refer to study designs where parents are asked questions such as "in the past two weeks has your child had an upper respiratory infection", rather than counting incident cases of infection over continuous periods of time.

4. heterogeneity test was lacking in this study. It should be included in methods and results section. If high heterogeneity was found, then additional analyses such as subgroup analysis or meta-regression could be performed.

We have revised the methods section to mention heterogeneity as follows: *"The I2 statistic was calculated as a measure of heterogeneity."* We did report the I2 statistic in Figure 2 with each meta-analysis and have updated the results section to include these figures within the written text. We do mention in the discussion that the heterogeneity appears to be quite high as per the I2 statistic, however, when visually examining the results, they do not appear very heterogenous. We believe that this is because our meta-analyses include registry studies which have very large sample sizes (and therefore narrow confidence intervals).

5. In introduction section, the necessity of conducting this meta-analysis (no synthesis of the research, p4, line 20) seems not suFicient.

We have added some additional justification highlighting that infection is one of the leading causes of morbidity in children as follows: *"Infection is a leading cause of childhood morbidity and a major cause of mortality in low and middle income countries,20 but there has been no comprehensive synthesis of the data regarding mode of birth and childhood infection that examines various levels of infection severity and comparing clinical infection categories. Synthesis of observational data is of particular importance in relation to mode*

of birth where a randomised controlled trial is unethical in most circumstances."
Minor comment :
6. In method section (P5), it might be more rational to put study search forward of study exclusion criteria.
Because we developed the study eligibility criteria prior to conducting the literature search we feel that it is clearer presented in the current format.
7. Page 6 line 22-24: why the four factors were selected as important confounders. Maternal BMI might be more important than maternal smoking during pregnancy.
We have now included our hypothesised causal model in our supplementary material to provide further information as to how we selected our important confounders. We agree that maternal BMI is closely linked with the exposure and was often satisfied our requirement of including 'an indicator for CS'. However there is less literature linking BMI with infection outcomes in children as compared to maternal smoking. Maternal smoking was considered an important confounder not only for its influence on maternal and child health but also as a proxy measure for more diFicult to measure factors such as socioeconomic position.
8. Page 13, line 41: please spell the full name of "ICD" when it was firstly presented.
Thank you for picking this up. We have made this change.
9. In discussion section, the OR 1.3 was diFerent from this study. The reason underlying this diFerence is needed to be mentioned.
There are a few possible reasons. First, the reporting of this study was very unclear and importantly they do not mention whether they used the adjusted odds ratios from the original studies or whether they calculated crude odds ratios from the aggregate numbers from the original studies to calculate the pooled odds ratio. Second, the odds ratio will typically be further from the null than a risk/hazard ratio where the outcome is not rare. Third, this study only synthesised three studies concerning respiratory infections. We have revised our manuscript text as follows: *"A previous systematic review of respiratory tract infections, among other paediatric outcomes, reported an odds ratio of 1·30 (95% CI 1·06-1·60) based on three studies, but did not diMerentiate between infections of the upper and lower respiratory tract and had unclear methodology on whether the crude or adjusted odds ratio was calculated.14"* However, we have kept this fairly brief as we do not want to give undue attention to this other meta-analysis which does not report their methodology suFiciently.

Reviewer 3
1. ABSTRACT
1.1 Data sources: the phrase inception is a little odd, please add the specific period of time to inform the reader.
We have changed "inception" to "no restriction on start date".
2. INTRODUCTION
2.1 Though the introduction is very short, it contains all the important background and objectives. I would stress some more that conducting this research by including observational studies is highly relevant since it is unethical to perform an RCT regarding this matter. Moreover, based on other RCT's which are more related to onset of labour, 70-90% of women decline participation and therefore this systematic review and metaanalyses are of great importance particularly due to the increase of CS in Western high income countries.
Thank you for this comment. We have included the following additional sentence in our introduction: *"Synthesis of observational data is of particular importance in relation to mode of birth where a randomised controlled trial is unethical in most circumstances."*
2.2 Could you elaborate a little more about reasons for women to get an elective or emergency CS? So the reader is more informed about the diFerences in the provision of either elCS or emCS.
In the context of this systematic review, it is not possible to elaborate on these important issues because the drivers of the decision to proceed to CS vary between settings (as reflected in the marked variation in CS rates) and the categorisation of CS as 'elective' and 'emergency' varies between sites, as we have addressed in the Discussion.
2.3 In the paragraph it is stated "examining diFerent levels of infection severity and clinical infections categories", these phrases do not further appear in the manuscript. Please make this consistent to prevent confusion.

Thank you for this suggestion. We have revised each section so that the description of the infection outcomes to be more consistent throughout.
2.4 The references can be also updated by adding some of the studies of Supplementary materials 2. Or are the references stated here from systematic reviews/meta-analyses only? Please make this more consistent or clear.
The references stated here were referring to previous systematic reviews. We apologise that this detail got lost at some point in our manuscript revisions and have now updated as follows: *"Mode of birth and infection has also been examined in previous systematic reviews in relation to specific types of infection"*
3. METHODS
The inclusion of studies regarding observational studies by including case control or cohort (follow-up or registry based) and excluding non-population based studies is very well written and clearly described.
Thank you.
3.1 MAJOR. My comment regarding the project is related to the modes of births in the study. The modes of births of interest are elective CS and emergency CS, these will be separately compared with vaginal births. These vaginal births are grouped with spontaneous or instrumental (forceps or vacuum extraction) vaginal births. Based on our own previous work we observed that compared with spontaneous vaginal births, also children born with instrumental vaginal births had higher odds of infections (respiratory, gastrointestinal and other infections, Peters et al. 2018). So I was wondering why did you not decide to conduct the analyses with all modes of births separately:
-Spontaneous vaginal birth (=reference)
-Instrumental vaginal birth
-Elective CS
-Emergency CS
Moreover, why did you not mention the results (ORs) of instrumental vaginal births in the supplementary table 2?
The choice to conduct our meta-analyses by emergency and elective CS was to ensure we included as many populations as possible in light of the diFerences between studies, which made synthesis challenging (e.g., exposure categorisation, infection outcome, eFect measure, follow-up age etc. as discussed in the methods section). We agree that these findings for more detailed categorisations of mode of birth are interesting and have included a further results paragraph to discuss the results presented in supplementary table 2, including the results on other mode of birth categorisations as follows: *"Some studies examined instrumental vaginal birth separately to non-instrumental vaginal birth and included birth interventions such as induction of labour in mode of birth categorisations. These studies generally reported higher risk with instrumental vaginal and induced births (both vaginal and caesarean section), albeit with some exceptions.36,38,48"*
3.2 MAJOR. This comment is related to the included population of interest. Did you include studies reporting results of term pregnancies only (≥ 37 weeks)? Or did you also include studies in the analyses with (very) pre term birth for which adjustments were made in the multivariable regression model? And if so: was gestational age an important confounder and therefore a requirement for inclusion? Otherwise, my concern will be that the (very) pre-term birth is highly associated with children's infections and not the mode of birth.
We excluded studies that only included preterm births based on the same concern that preterm birth is highly associated with childhood infection. We included studies where the results considered preterm and term births together. Although we did not require gestational age as one of our important confounders (partly because of the ongoing debate as to whether it is better conceptualised as a confounder or mediator in relationship to mode of birth), we note that: (1) most studies did adjust for gestational age where both preterm and term births were included and (2) where no distinction is made between preterm and term, the contribution of preterm births will be relatively small given the proportion of births they constitute. We have added the following sentence discussing this aspect: *"In our risk of bias assessment, we did not require adjustment for gestational age as one of our important confounders because it may be considered conceptually as a confounder or a mediator in diMerent scenarios. However, all but four studies39,45,49,59 accounted for gestational age through statistical adjustment, restricting analyses to term pregnancies, or including sensitivity analyses."*
3.3 The categorization of the outcomes (i.e. infections) is quite detailed with four diFerent

categories. However, throughout the manuscript there is no mentioning on these categories since the infection categories are broadly described (hospital versus nonhospital/ other infections). Please make this consistent throughout the manuscript to enhance clarity.
We have made changes throughout the manuscript to make our infection categorisation more consistent/clear. In particular we have added the following detail to mention where these four clinical infection categories arise: *"As a result of this hierarchy, we were able to perform meta-analyses using the reported hazard ratios for elective/planned caesarean section and emergency/acute caesarean section separately for four hospitalised infection outcomes: overall hospitalised infections and three common clinical infection categories (upper respiratory, lower respiratory and gastrointestinal infections)."*
3.4 In the statistical paragraph you state: "Due to overlapping study populations across studies, we considered several study features to determine which results to statistically synthesise in meta-analyses according to the following hierarchy (….) (iii) similarity of the eFect measure type (e.g., odds, hazard, or risk ratio) as the odds ratio is not an appropriate estimate of the risk ratio for non-rare outcomes" What is the diFerence between the odds and the odds ratio? Do you mean something diFerently? As an epidemiologist I do agree that reporting the HR is more preferred than the OR. However, can you back this statement up with a reference as well?
When we discuss considering the similarity of the eFect measure type, it is not that we preferred the hazard ratio over the odds ratio from the outset but more that the two measures are not comparable for non-rare outcomes, and it is inappropriate to statistically synthesise them together. We therefore looked for similarity in the eFect measure across the available studies with HR being the most commonly used eFect measure.
3.5 MAJOR. Concerning the outcome and the time that an infection can occur, is it very relevant to have such long-term follow-up period of the children? Particularly the studies reporting HRs up till 18 years of age. Many other factors may have contributed to particular infections? In the study protocol in Prospero there is mentioned that subgroup analyses (e.g. age, gender, country income level) will also be performed, did you perform a subgroup analysis for age-groups?
We agree that the length of follow-up is important and that postnatal factors will have increasing eFect on infection risk as children age. However, we decided to include all the studies that look at childhood in preference to choosing an arbitrary cut-oF age for the purpose of our review. We had hoped to do sub-group meta-analysis by age of follow-up when we registered our protocol but unfortunately there were insuFicient studies to do this (which was of course not apparent prior to the literature review). We have included additional detail in the results section for the included studies which had sub-group analyses by follow-up age as follows: *"There were too few studies to explore follow-up age in our meta-analyses. In five studies26,33,46,57,58 including sub-group analyses by age, there was a small attenuation with longer follow-up, but this was not consistent nor particularly pronounced."*
3.6 The studies reporting ORs in this field of research are also very relevant and important information, which you also show in supplementary table S2. This is highly appreciated since you show results for the HRs and ORs regarding the study aim. Thank you for being so thoroughly.
Thank you.
4. RESULTS
The results are very objectively and clearly written.
4.1 Regarding Table 1, I would adapt the title by informing the reader from the beginning if the mentioned studies included hospital infections an non hospital infections. Also, in the table the main exposure of interest (i.e. modes of birth) is reported, I observed that only the study of Barnes 2019 included instrumental vaginal birth? Is this also the reason why you have grouped them together (see also previous comment)?
In Table 1 there are two main banners that signify whether the study includes hospitalised or other/non-hospitalised infections as the outcome. We have revised the supplementary material to include the results where more detailed mode of birth categories were used beyond emergency/elective including results for instrumental and non-instrumental vaginal births. We chose not to report all these vaginal birth categories in Table 1 and instead list only the reference category. This was to keep the table at a manageable size and instead focus on the CS categorisations here as they relate to our main research

question. The Barnes 2019 study has instrumental vaginal listed because that was their reference category, but it was not the only study to look at instrumental vaginal birth.
4.2 In table 1 diFerent sample sizes are included from studies with small (n=288) and large groups (=> 1 million). Are these diFerent sample sizes influencing your results? Do you have to take this in to account in the statistical analysis?
Yes, the meta-analysis will give more weighting to studies with larger sample sizes. Although, the variation in sample size within the meta-analysed studies is less pronounced (as they were all registry-based studies) than the diFerences noted in Table 1 which shows all the studies included in the review as a whole.
4.3 MAJOR. Regarding the manuscript, hospitalised infections. In this paragraph it is mentioned why some studies are in/excluded in the meta-analyses. The phrase overlapping populations is triggering me since are these populations really overlapping or did they select diFerent pregnancies e.g. all pregnancies versus healthy pregnancies? Moreover, maybe some studies were also excluded because these reported ORS (Peters et al) and therefore results were shown in Supplementary Tables? Also there is more information shown in Figure 1 Flowchart, please make this more consistent since this is really relevant to know as a reader.
The overlapping populations vary in their degree of overlap depending on the particular years covered by each individual study and diFerences in how they define the study population (e.g., all pregnancies versus healthy pregnancies, as the reviewer highlights). The overlapping populations did typically arise from more than one registry-based study on the same population and so we do think that with total population data (in a registry-based study) that this is substantial enough overlap that it would be statistically inappropriate to synthesise the results together. The Figure 1 flowchart exclusions refer to studies that are excluded from the review altogether rather than from meta-analyses.
4.4 Regarding Figure 1 (flowchart)
Please add an ‘exclusion box’ between 5398 title and abstracts screened and 124 full text assessed for eligibility.
Please consider to add two additional boxes under 31 studies included for:
Hospital infections (including number of studies) and non-hospitalised infections (including number of studies) and could you add the range of period of time as well for the included studies?
We have made the suggested changes to Figure 1.
5. DISCUSSION
5.1 MAJOR. The discussion includes all relevant sections (strengths, limitations, recommendations etc). I really appreciate the paragraph about the confounder by indication, since this is a major concern which has been very well addressed by the authors. It would suggest to elaborate some more about the total trajectory of labour and birth regarding other factors that are contributing to increased risk of infections (e.g. induction of labour, gestational age, and long-term follow-up period).
These are important points which we hope that we have discussed in suFicient detail, mindful of the need for balance between being relatively concise but also addressing the major issues.
5.2 MAJOR. It would be valuable to have some reflection on the registration of infections across countries. For example, infections are very well registered in Australia and perhaps therefore there is a very high prevalence on infections in secondary care. Furthermore, there might be data classification issues regarding the classification on elective CS and emergency CS in electronic health registry data. These data are primarily for routine care and secondary for research which may cause some misclassification.
Thank you for raising this important point regarding the secondary use of registry data. We have included the following sentence in the discussion to address this data limitation:
“Similarly, most studies on hospitalised infections used diagnoses from hospital registries coded using the International Classification of Diseases (ICD). However, there may be some misclassification of diagnoses as these data are primarily collected for administrative purposes rather than for research. It is possible that the parameters used when assigning ICD-coded discharge diagnoses may vary between settings.”
5.2 MAJOR. The reflection on results (comparison similar research) is based on OR’s instead of HRs. Why did you not compare your results with HR, particularly since the OR’s mentioned are part of the supplementary materials. Moreover, you made quite a statement in the statistical paragraph (methods) about your preference for HR.

We mention this OR in the comparison with similar research because it is drawn from a systematic review and to elaborate on what prior research has found on the topic (rather than a direct comparison of the HR from our meta-analyses with the OR in this case). We have included further discussion of the findings shown in the supplementary material in our revised manuscript as per previous responses.

5.3 The phrase about causality (page 14, sentence 33-34) is not correct since it is impossible to infer causality from the results of observational studies.

We are aware there are diFerent viewpoints on the use of causal language in relation to observational studies. We share the opinion of some others (doi:10.1001/jama.2024.7741) that it is reasonable to state causal hypotheses as they inform the study rationale and make some causal inferences in certain circumstances, but that the results of observational studies should not be presented using causal language.

5.4 I would reflect some more about the bias in the included studies, particularly since you have created a very nice overview presented in the supplementary materials.

Thank you for this comment. We have included the overall risk of bias ratings in the revised text at the beginning of paragraph 2 of the results section as follows: *"The overall risk of bias assessment was 'low' for 5 studies,31,33,38,42,43 'some concerns' for 15 studies,26,30,32,34,36,44-47,51-54,56,59 'high' for 8 studies,35,37,39,41,48,49,57,58 and 'very high' for 3 studies.40,50,55"*

5.5 It would be very worthwhile to reflect some more about the values of the HRs for clinical practice. What could be the practical implication for this in daily care?

Given that this is an observational study across many diFerent settings, we are cautious in being too explicit about clinical implications that may not be generalisable.

5.6 The impact of CS on infections is also reflected with the microbiome however it is lacking the potential interaction with epigenetics eFects as well. Please see this paper and consider to add this as well from Dahlen et al 2016: Childbirth and consequent atopic disease: emerging evidence on epigenetic eFects based on the hygiene and EPIIC hypotheses - PubMed (nih.gov)

While this paper relates more to atopic disease than infection, it is true that they may share some similar biological pathways through impacts on immune responses, so we have included the following sentence to reflect the possibility of epigenetic eFects as well: *"Other mechanistic theories point to the potential impact on immune response of epigenetic alterations following caesarean section birth and associated intrapartum interventions.22"*

5.7 Finally, your last paragraph you state: across a variety of settings? What do you mean by this? Several high income countries? A variety of settings, opens a whole new box for me, for example I think about several maternity care settings. Which is in my opinion not what you want to phrase here, please be scientifically objective as you have perfectly done throughout the manuscript.

Thank you for this feedback. We were trying to convey the consistency across several highincome countries and diFerent cohorts. We have removed the phrase "across a variety of settings."

Reviewer 4

Comments:

The present systematic review and meta-analysis was performed to synthesize the evidence on the risk of hospitalised and non-hospitalised infections in children born by Csection compared to children born by vaginal birth. The review is well planned and conducted and the manuscript is very well written. The authors may wish to consider the following comments:

In the review protocol, the authors planned to include cohort, case-control or crosssectional studies. In the eligibility criteria section of the manuscript, they only listed cohort or case-control studies. Although it is understandable that cross-sectional studies may not be eligible if they were done to estimate the prevalence rather than an association, some retrospective cohort studies, or even longitudinal cohort studies are sometimes described as cross-sectional studies. Cross-sectional studies are included in the search strategy, which is the right thing to do but the authors should clarify this point in this section as to how they handled this issue.

Thank you for this suggestion. We had removed cross-sectional from this section of the

methods text when we clarified that we wanted studies that estimated incident rather than prevalent cases of infection. However, we agree with the reviewer's comment that some retrospective or longitudinal studies may be described as cross-sectional. We have therefore revised the text in the methods to include cross-sectional studies (as per the protocol) but kept the exclusion criteria of incident rather than prevalent cases of infection as is. We hope this clarifies this point.

The authors searched PubMed and Embase but they should have searched Web of Science to ensure a comprehensive search.

These aspects are always a balance between ensuring a comprehensive search and having a manageable number of studies. We consulted with a senior health librarian to determine which databases to search and searched three databases as well as forwards/backwards citation searching of the retrieved articles. We also ensured that our search identified all of the articles of which we had prior knowledge. Therefore we believe that the search was suFiciently comprehensive.

Did IT and JM do the data extraction independently? I assume two review authors did the data extraction but this should be stated.

IT extracted the data and JM checked the extracted data. We have clarified this in the manuscript methods section as follows *"Data from each study were extracted by one reviewer (IT) and verified by a second reviewer (JM) using a piloted form which collected information on the study population, design, statistical analysis, and findings."*

The authors pre-specified the key potential confounders in the risk of bias assessment. They listed maternal age, smoking, socio-economic status and "a maternal risk factor for caesarean section". Do they mean just one risk factor for C-section? Which factor is this? Or do they mean any risk factor for C-section? There is a long list of risk factors for Csection and the authors should be clear here.

We are sorry that this was not clear. We meant at least one risk factor for C-section so that it was not overly stringent in requiring a specific risk factor. We have included our hypothesised causal model as a supplementary figure (in response to previous comment) and highlighted some common CS risk factors that met this criterion. We have also revised the text to clarify the key potential confounders as follows *"Based on our causal model (**Supplementary Figure 1**) we pre-specified four factors – maternal age, maternal smoking during pregnancy, socioeconomic status, and at least one maternal pregnancy or healthrelated risk factor for caesarean section – as important confounders requiring adjustment, in the preliminary assessment of the ROBINS-E tool."*

Does ROBINS-E provide an overall score or another formula that enables researchers to classify study into low, moderate or high risk of bias?

Yes, ROBINS-E has an overall score of the risk of bias which is typically the highest domain level risk of bias. These study-level overall scores are shown in the supplemental figure. We have also included these overall scores in the revised manuscript at the beginning of paragraph 2 in the results section as follows: *"The overall risk of bias assessment was 'low' for 5 studies,31,33,38,42,43 'some concerns' for 15 studies,26,30,32,34,36,44-47,51-54,56,59 'high' for 8 studies,35,37,39,41,48,49,57,58 and 'very high' for 3 studies.40,50,55"*

Considering that all eligible studies are observational, what was the highest score that can be allocated to each study? Were all studies downgraded because they are observational, rather than RCTs, or all studies started at the highest grade? Some detail about the factors that led to downgrading would be helpful. Also, most of the tables and figures in the appendix are presented in a way that is hard for reviewing on a computer.

Referring to the GRADE framework, the certainty of evidence begins at a level of low when the findings are based on observational studies and can be upgraded for the following reasons: (1) large magnitude of eFect, (2) all plausible confounding would reduce the demonstrated eFect or increase the eFect if no eFect was observed, (3) dose-response gradient. Our findings did not satisfy either criteria 1 or 2 for upgrading and criteria 3 does not apply to our exposure and therefore were not upgraded from this initial level of 'low' certainty. Other considerations for downgrading are listed in the supplemental figure.

On page 14, line 8, the authors state "a randomised controlled trial of mode of birth is clearly unethical". Although I understand the authors' rationale for making this statement, I am not sure if there were evidence to back it up. It is understood that randomising women to have a C-section without medical indication is contentious, but saying that it is unethical should be backed by evidence.

Thank you for this comment. We have softened the language here as there are some circumstances where an RCT of mode of birth may be considered appropriate. The revised sentence reads: *"The certainty of evidence under the GRADE framework reflects that all the studies were observational; a randomised controlled trial of mode of birth would be unethical in most circumstances."*

Considering the small magnitude of the reported estimates, and the potential unmeasured confounding, the authors should take these issues into account in their conclusions. Previous studies on C-section and child morbidity found that some of the reported associations are related to unmeasured familial confounding rather than causal associations. This is important considering that the authors argue that these results "should inform the design of mechanistic studies and intervention trials…". If the observed associations are not causal they may end up misleading the design of future studies, especially intervention trials. Also, the authors suggest that breastfeeding may partially explain the eFect of C-section on the risk of infections in the child. Shouldn't breastfeeding be considered a potential mediator rather than a confounder and therefore it is not explain the association in terms of causality but rather mediate the association?

Thank you for this comment. We agree that this is a potential issue with the modest eFect size and potential confounding that we discuss within the manuscript. We have changed this sentence to read "*may inform*" rather than "*should inform*". We were trying to convey here that epidemiological studies are the starting point to inform mechanistic studies, which in turn may guide interventions and policy.

With regards to your comment on breastfeeding being considered a mediator, we agree that breastfeeding is a potential mediator rather than a confounder. By 'explain the eFect' here we mean that because CS is associated with lower breastfeeding levels, then it could be through this potential mediating pathway that we are seeing a CS -> infection association rather than a direct eFect of CS itself. We have revised the text as follows to make this clearer: *"Mediation through early life factors may also partially explain the eMect. For example, breastfeeding reduces the risk of childhood infection67 and breastfeeding initiation is generally lower following caesarean section birth.68"*

VERSION 2 – REVIEW

**REVIEWER NAME**	Riley, Richard
**REVIEWER AFFILIATION**	University of Birmingham, Institute of Applied Health Research
**REVIEWER CONFLICT OF INTEREST**	None
**DATE REVIEW RETURNED**	23-Aug-2024

**GENERAL COMMENTS**	The responses to previous comments seems appropriate and the revision has improve the paper. In particular, I checked the responses to the previous statistical reviewer and these were appropriate. I have been through the revised article myself and have a few comments for additional improvement.<br><br>1) Abstract: results should mention the range of median/mean follow -up lengths across the included studies, to get a feel for the lengths of follow-up of the included studies<br>2) Abstract: there is no mention of the heterogeneity of the study effects in the meta-analysis, or the RoB conclusions, or the GRADE conclusions.<br>3) The authors use the DeSimonian and Laird method for estimation of the random effects model (ideally REML is preferred: https://pubmed.ncbi.nlm.nih.gov/30067315/), but do not state how CIs are derived. Evidence suggests the Hartung Knapp correction is preferred. https://pubmed.ncbi.nlm.nih.gov/30067315/ - likely to give wider CIs, so please consider this as a sensitivity analysis at least<br>4) I wonder if there was any evidence (in the original studies) that the HR was not a constant over time? That is, different studies have different lengths of follow-up, and the HR might perhaps wane over

	time. Was this considered at all? Something to discuss at least.<br>5) The authors report I-squared as a 'measure of heterogeneity' – however, it does not actually measure heterogeneity, but the proportion of the total variance of effect estimates due to heterogeneity. This needs to be explained better. Rather, the authors should (also) report tau-squared (or tau), the estimate of between-study heterogeneity (standard deviation) for each of the meta-analyses.<br>6) To disseminate heterogeneity even more clearly, the authors might also consider reporting 95% prediction intervals. https://www.bmj.com/bmj/section-pdf/186285?path=/bmj/342/7804/Research_Methods_Reporting.full.pdf - in particular, this would help show that most meta-analysis have heterogeneity but that the range is still in the area of HRs > 1<br>7) In the main results section, please add the number of studies alongside each meta-analysis result<br>8) The authors refer to an increased 'risk' when interpreting the HRs, but technically are they more correct referring to an increased hazard (or rate)? For example, 'Those born by elective caesarean had an estimated 16% increased risk of hospitalised upper respiratory infections' – I think 16% increased hazard rate is more correct?<br>9) I think the forest plots should also include the % study weights, to help reveal the contribution of each study<br>10) Crucially, we do not see (perhaps I missed it?) the adjustment factors used in each study contributing to each meta-analysis. I think this needs greater clarity.<br><br>I look forward to seeing the revision in due course, and I hope my comments are constructive for the authors moving forwards, in the context of clear hard work and good research paper.

VERSION 2 – AUTHOR RESPONSE

Reviewer comments:
1. Abstract: results should mention the range of median/mean follow-up lengths across the included studies, to get a feel for the lengths of follow-up of the included studies.

Response: We have updated the following sentence in our abstract to give the range of follow-up lengths: "Cohort sizes ranged from 288 to 7.2 million and follow-up age from one to 18 years."

2. Abstract: there is no mention of the heterogeneity of the study effects in the meta-analysis, or the RoB conclusions, or the GRADE conclusions.

Response: Thank you for this suggestion. We have updated the abstract to include the heterogeneity statistics alongside each meta-analysis result and have also included the following sentence regarding risk of bias conclusions: "Risk of bias concerns primarily related to confounding." We felt that the abstract was already lengthy and that the GRADE conclusions need to be appropriately contextualised within the GRADE framework (with the default rating of observational evidence as 'low'). Therefore, we did not add these conclusions to the abstract, but we do acknowledge the important limitation of potential confounding in our abstract conclusion.

3. The authors use the DeSimonian and Laird method for estimation of the random effects model (ideally REML is preferred: https://pubmed.ncbi.nlm.nih.gov/30067315/), but do not state how CIs are derived. Evidence suggests the Hartung Knapp correction is preferred.
https://pubmed.ncbi.nlm.nih.gov/30067315/ - likely to give wider CIs, so please consider this as a

sensitivity analysis at least

Response: Thank you for this suggestion. We have updated our meta-analyses to use the REML estimator for tau2 and the Hartung Knapp correction for our confidence intervals. The updated text in the methods reads as follows: “Meta-analyses used a random-effects model and the restricted maximum likelihood (REML) estimator of τ^2 to estimate the pooled hazard ratio.29 Confidence intervals and prediction intervals were derived using the Hartung-Knapp method.30 31”

4. I wonder if there was any evidence (in the original studies) that the HR was not a constant over time? That is, different studies have different lengths of follow-up, and the HR might perhaps wane over time. Was this considered at all? Something to discuss at least.

Response: Yes, we did consider this and went over the studies where different HRs were reported for the same population with different follow-up ages. The following sentences in the results section on page 11 reflects what these studies showed: “There were too few studies to explore follow-up age in our meta-analyses. In five studies26,36,49,60,61 including sub-group analyses by age, there was a small attenuation with longer follow-up, but this was not consistent nor particularly pronounced.”

5. The authors report I-squared as a ‘measure of heterogeneity’ – however, it does not actually measure heterogeneity, but the proportion of the total variance of effect estimates due to heterogeneity. This needs to be explained better. Rather, the authors should (also) report tau-squared (or tau), the estimate of between-study heterogeneity (standard deviation) for each of the meta-analyses.

Response: We have updated our results to include tau2 alongside I2 in the text rather than only in the figures (as it was previously). We have also revised the manuscript sections as follows:

Methods: “For each meta-analysis we report the τ^2 and I^2 statistic as measures of the between-study variance and the proportion of total variance attributable to between-study heterogeneity respectively.”

Discussion: “I2 is commonly used as a statistical measure of heterogeneity and was considered moderate to high in our meta-analyses. However, I2 is the proportion of the total variance that would remain if the variance due to sampling error is removed.64 Because our meta-analyses included studies with large sample sizes and corresponding high precision, the variance due to sampling error component will be small. Therefore, the high I2 values mainly reflect the precision of the included studies rather than large observable heterogeneity in the results.”

6. To disseminate heterogeneity even more clearly, the authors might also consider reporting 95% prediction intervals. https://www.bmj.com/bmj/section-pdf/186285?path=/bmj/342/7804/Research_Methods_Reporting.full.pdf - in particular, this would help show that most meta-analysis have heterogeneity but that the range is still in the area of HRs > 1

Response: Thank you for this suggestion. We have updated our meta-analysis figure to include the prediction interval for each result and updated the manuscript text as follows:

Methods: “Confidence intervals and prediction intervals were derived using the Hartung-Knapp method.30 31”

Discussion: “The calculated prediction intervals illustrate that although there was some variation in study estimates, the direction of effect was consistent.”

7. In the main results section, please add the number of studies alongside each meta-analysis result.

Response: We have updated our results text to include this.

8. The authors refer to an increased 'risk' when interpreting the HRs, but technically are they more correct referring to an increased hazard (or rate)? For example, 'Those born by elective caesarean had an estimated 16% increased risk of hospitalised upper respiratory infections' – I think 16% increased hazard rate is more correct?

Response: While the hazard ratio is commonly interpreted as 'risk' we have updated the manuscript text where we are specifically referring to the pooled hazard ratio from meta-analyses to read, for example: "Those born by elective caesarean had an estimated 16% increased rate of hospitalised upper respiratory infections" to be more technically correct as suggested.

9. I think the forest plots should also include the % study weights, to help reveal the contribution of each study

Response: We have now included this in the forest plots.

10. Crucially, we do not see (perhaps I missed it?) the adjustment factors used in each study contributing to each meta-analysis. I think this needs greater clarity.

Response: We included the adjustment factors for each individual study in Table 1 (now moved to supplementary Table 1). To give greater attention to this we have revised the following sentence: "Individual study assessments for risk of bias are shown in Supplementary Figure 2 and confounder adjustments for each study are listed in Supplementary Table 1."
